# Metabolomic Profiling of the Host Response of Tomato (*Solanum lycopersicum)* Following Infection by *Ralstonia solanacearum*

**DOI:** 10.3390/ijms20163945

**Published:** 2019-08-14

**Authors:** Dylan R. Zeiss, Msizi I. Mhlongo, Fidele Tugizimana, Paul A. Steenkamp, Ian A. Dubery

**Affiliations:** Centre for Plant Metabolomics Research, Department of Biochemistry, University of Johannesburg, P.O. Box 524, Auckland Park, Johannesburg 2006, South Africa

**Keywords:** chemometrics, LC-MS, metabolite profiling, metabolomics, *Ralstonia solanacearum*, Solanum lycopersicum

## Abstract

Tomato (*Solanum lycopersicum*) is an important dietary source of bioactive phytochemicals and active breeding programs constantly produce new cultivars possessing superior and desirable traits. The phytopathogenic *Ralstonia solanacearum*, the causal agent of bacterial wilt, is a highly destructive bacterial disease with a high economic impact on tomato production. This study followed an untargeted metabolomic approach involving four tomato cultivars and aimed at the identification of secondary metabolites involved in plant defense after infection with *R. solanacearum*. Liquid chromatography coupled to mass spectrometry (LC-MS) in combination with multivariate data analysis and chemometric modelling were utilized for the identification of discriminant secondary metabolites. The total of 81 statistically selected features were annotated belonging to the metabolite classes of amino acids, organic acids, fatty acids, various derivatives of cinnamic acid and benzoic acids, flavonoids and steroidal glycoalkaloids. The results indicate that the phenylpropanoid pathway, represented by flavonoids and hydroxycinnamic acids, is of prime importance in the tomato defense response. The hydroxycinnamic acids esters of quinic acid, hexoses and glucaric acids were identified as signatory biomarkers, as well as the hydroxycinnamic acid amides to polyamines and tyramine. Interestingly, the rapid and differential accumulation of putrescine, dopamine, and tyramine derivatives, along with the presence of a newly documented metabolite, feruloyl serotonin, were documented in the infected plants. Metabolite concentration variability in the different cultivar tissues point to cultivar-specific variation in the speed and manner of resource redistribution between the host tissues. These metabolic phenotypes provide insights into the differential metabolic signatures underlying the defense metabolism of the four cultivars, defining their defensive capabilities to *R. solanacearum*.

## 1. Introduction

Tomato (*Solanum lycopersicum*), from an economic standpoint, is one of the most important horticultural crops cultivated [1,2]. However, global tomato production and yield is plagued by several diseases caused by a number of pathogens. One of the major challenges for a rapid growing global population is the need to meet the demand for adequate food supply. This requires ecologically sound, compatible strategies in agriculture for sustainable crop production. However, emerging and re-emerging plant diseases continuously challenge this task, consequently, the management of plant diseases continues to be an uphill task [3]. Solanaceous crops, especially tomato, serve as excellent model systems for the investigation of plant-pathogen interactions [4]. 

*Ralstonia solanacearum* (*R. solanacearum*), formerly classified as *Pseudomonas solanacearum*, is a gram-negative phytopathogenic β-proteobacterium that can infect more than 450 plant species in approximately 50 botanical families throughout the world, but seems to be particularly pathogenic to the eudicot Solanaceae family [5,6,7,8]. As the causal agent of bacterial wilt (BW) disease in tomato, *R. solanacearum* is among the most prevalent plant pathogens, and has caught the world’s attention due to its destructive capacity, unusually wide host range, persistence and broad geographical distribution [9,10,11,12]. The broad genotypic and phenotypic variation reflected within the heterogeneous species has led to the emergence of the pathogen being described as the *Ralstonia solanacearum* species complex (RSSC) [6,13,14].

As a typical soil-borne pathogen, the *R. solanacearum* bacterium invades a suitable host plant by gaining access to the root system via wounding, root tips or cracks produced at sites of lateral root emergence [7,9,13]. Upon entry, the pathogen colonizes the root cortex, then subsequently, invades the water-transporting xylem channels to facilitate migration between the root and shoot systems by means of the vascular parenchymal bundles [5,7]. To establish itself inside the host plant, and ensure effective multiplication within the plant tissues, the pathogen must be metabolically proficient and deploy energy to set up the necessary elements required for the infection process [13]. Once established, the bacteria multiply to high cell-densities and produce several pathogenicity determinants [5,15]. Pathogenicity factors produced during infection that contribute to pathogen virulence and metabolic proficiency include the type III secretion system (T3SS), core effector molecules, several cell wall degrading enzymes and exopolysaccharides (EPS) [5,8,16,17,18]. As the pathogen multiply to high cell-densities, EPS production also increases, which has been suggested to result in a physical blockage of the xylem vessels. This prevents water distribution to the upper organs of the plant, resulting in generalized wilting of the leaves and stems followed by total plant death [5,7,10,16]. This method of infection has made *R. solanacearum* a model organism for studying plant-pathogen interactions involved in colonization of the water-conducting xylem tissue, a nutrient-poor and oxygen-deprived environment [16,19]. The economic impact of *R. solanacearum* has been difficult to quantify as a result of its global distribution and continually expanding host range [20]. Direct yield losses by *R. solanacearum* vary according to a number of factors including the host, cultivar, climate, soil type, cropping pattern, and strain—where crop losses in tomato can vary from 0% to 90% [21]. 

As the newest addition to the systems biology approaches, metabolomics has flourished and is now firmly established as the most downstream level of the “-omics” technologies, reflecting the phenotypic variation and contributing to an added dimension of understanding the biochemistry and molecular complexities of life [22]. Since its conception, metabolomics has rapidly developed over the last few decades, owing to the improvement in liquid chromatography linked to mass spectrometry (LC-MS) which has vastly improved in sensitivity, high throughput and metabolite coverage [23,24].

The metabolome of an organism encompasses the entire complement of small molecules, that directly reflect gene regulation leading to the production of these metabolites [25]. Metabolomics is thus defined as the comprehensive, qualitative, and quantitative study of all metabolites (≤ 1500 Da) in a cell, tissue or whole organism during certain environmental and genetic perturbations, to provide a biochemical description of the phenotypic status [26]. Metabolomics approaches have been successfully applied to various research fields including environmental and biological stress studies, functional genomics, biomarker discovery, biotechnology and integrative systems biology [27,28]. This adds to the understanding of biochemical fluxes and discoveries of metabolites, which are indicative of pathogen, pest or environmental perturbations [24,29]. It is seen as a data-driven approach associated with the unbiased assessment of all small molecules within a biological matrix, which separates it from traditional targeted phytochemical approaches. The measurements of the intracellular metabolites in the system yield insights into the metabolic changes that occur at cellular tissue and organismal level.

## 2. Results

### 2.1. Cultivar Information

The cultivars investigated, STAR9001 (1R), STAR9006 (6R), STAR9008 (8S), and STAR9009 (9S) were released from a tomato breeding program (Stark Ayres, Pty. Ltd. Bredell, South Africa). According to Starke Ayres, the 1R and 6R cultivars have been classified as exhibiting high resistance to *R. solanacearum*, while the 8S and 9S cultivars, exhibit intermediate resistance/tolerance to the pathogen ([30], Table S1). The disease severity index (DSI) was set up to monitor the progression of each cultivar’s symptoms associated with *R. solanacearum* infection. It should be noted that all cultivars investigated in this study are described as exhibiting an intermediate to high level of resistance to *R. solanacearum* but may exhibit variability in performance under certain environmental conditions and different geographical locations. Previous research indicated that the four cultivars showed a natural degree of variability in their underlying metabolic profiles which may alter their ability to effectively respond and fend off pathogen infection [30]. 

### 2.2. Symptoms and Characterization

Symptoms associated with *R. solanacearum* treatment included the generalized wilting of the upper leaves, along with the brown discoloration on the lower stem (Figures S1 and S2). All the cultivars exhibited extreme leaf wilting beyond recovery (Figure S2a,b). A cross-section through the stem of the treated cultivars revealed the presence of a milky-white exudate originating from the stem vascular bundles which was collected and plated on triphenyl tetrazolium chloride (TTC) medium and selective South Africa-Elphinstone (SMSA-E) media shown in Figure S2c,d. Colonies formed on the media exhibited a white margin with a pink or light red centralized color as well as a fluidal or mucoid morphology indicative of a virulent *R. solanacearum* strain [31,32,33]. A tomato cultivar was infected with the *R. solanacearum* strain isolated using the SMSA-E media to prove Koch’s postulates and validate the first infection (Figure S2e,f). The disease severity of each cultivar was monitored and scored based on the index criteria where the scoring ended on day 15, where all cultivars exhibited pronounced symptoms (Figure S3). The 6R cultivar demonstrated the slowest development of definite symptoms to *R. solanacearum*, typically associated with that of tolerant/ resistant cultivars, followed by the 1R and 8S cultivars and finally, the 9S cultivar which was the most susceptible to the pathogen.

### 2.3. Ultrahigh-performance Liquid Chromatography Coupled to Mass Spectrometry

**Figure 1:**
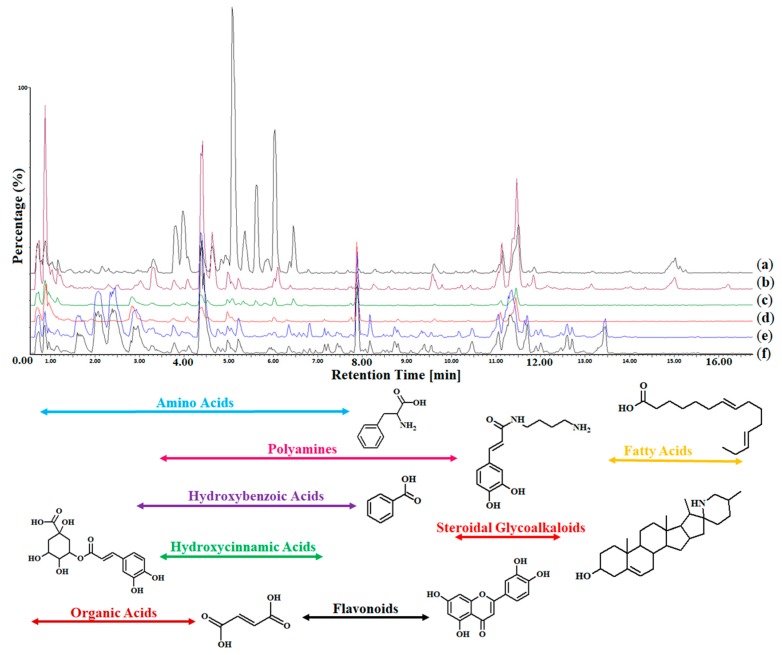
A representative ultrahigh-performance liquid chromatography-mass spectrometry (UHPLC-MS) base peak intensity (BPI) chromatogram (ESI(−) mode) overlay showing the metabolite profiles of tissues from the *Solanum lycopersicum* cultivar “1R” before and after treatment with *R. solanacearum.* (**a**) The 1R treated root sample. (**b**) The 1R control root sample. (**c**) The treated 1R stem sample. (**d**) The 1R control stem sample. (**e**) The 1R treated leaf sample. (**f**) The 1R control leaf sample. The y-axis represents the relative abundance (%) of the metabolite fragments at their respective retention times (min). A secondary metabolite belonging to each metabolite class is shown below the BPI overlay.

**Figure 2:**
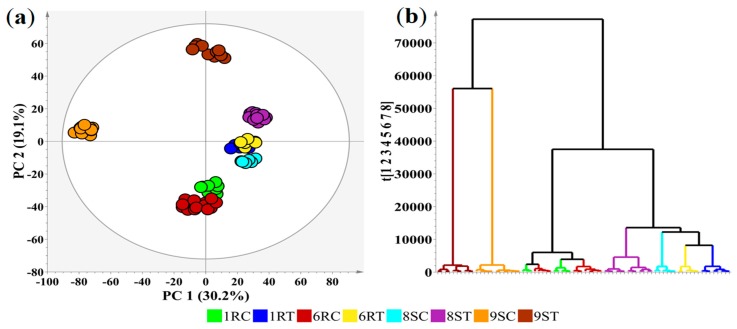
A PCA score plot of the UHPLC-MS ESI(−) data of the extracts prepared from the leaves of four *Solanum lycopersicum* cultivars treated with *Ralstonia solanacearum*. The labels C and T refer to the control and treatment of the four cultivars (1R, 6R, 8S, and 9S) with varying resistance (R) or susceptibility (S) to the pathogen. (**a**) A 2D principal component analysis (PCA) score plot illustrating the grouping of the variable conditions. The ellipse on the score plot represents Hoteling’s T2 with a 95% confidence interval. (**b**) A single-linkage hierarchical cluster dendrogram corresponding to (**a**), showing the hierarchical outline of the leaf data of the cultivars before and after treatment.

**Figure 3:**
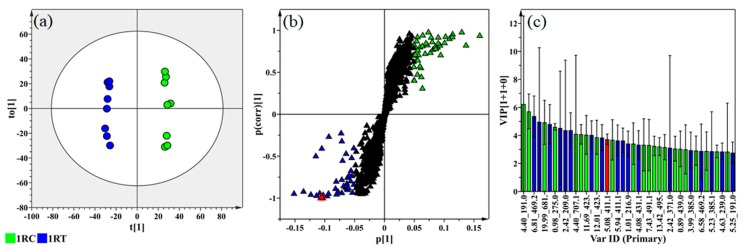
The selection of discriminant biomarkers, present in ESI(−) leaf tissue extract data, associated with the response produced by the tomato “1R” cultivar following infection with *Ralstonia solanacearum*. (**a**) An orthogonal projection to latent structures-discriminant analysis (OPLS-DA) plot showing the group separation of control vs. treated (1RC—Green vs. 1RT—Blue) conditions. (**b**) The corresponding OPLS-DA loading S-plot showing the targeted variables, with significant ions present in the lower left quadrant of the loadings S-plot (Blue) being positively correlated to pathogen treatment. Mass ions present in the upper right quadrant (Green) are negatively related to pathogen treatment and positively correlated with normal plant homeostasis. (**c**) The variable importance in projection (VIP) plot, for the model shown in (**a**), shows the mass ion *m/z* 411.184 taken as an example, annotated as caffeoyl putrescine (highlighted in red in b and c) in Table S2. Significant ions with VIP scores >1.00 were selected for further analysis.

**Figure 4:**
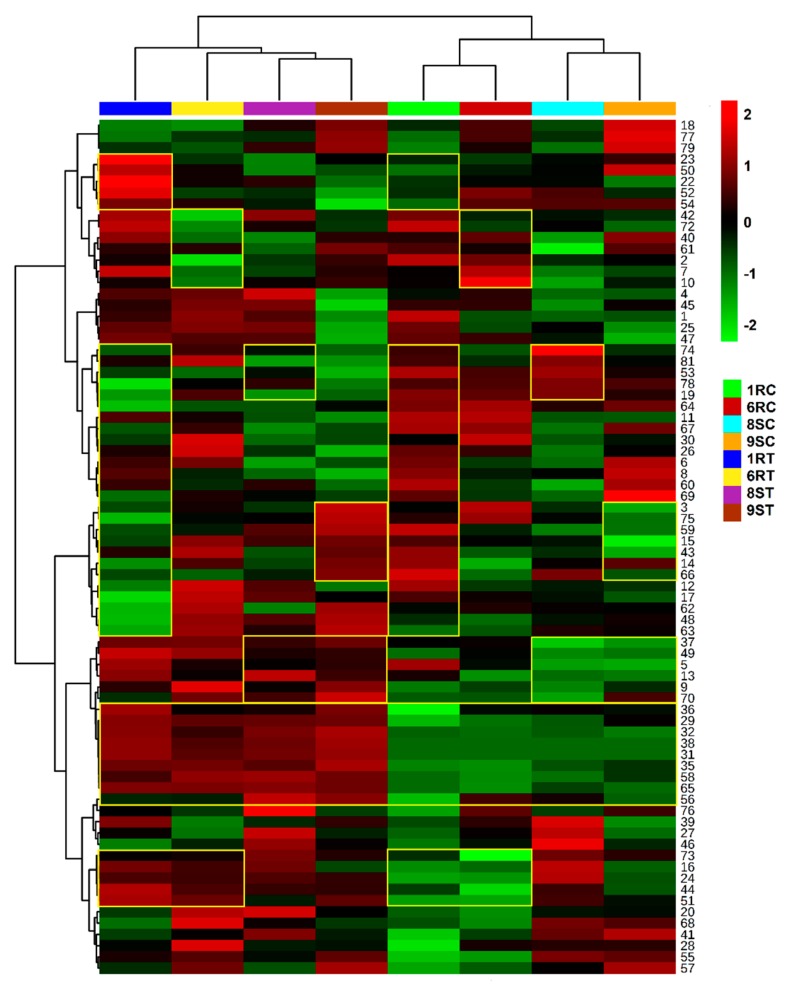
A heatmap analysis (Pearson distance and Ward’s linkage rule applied) showing the individual peak intensities of the 84 differential metabolite ions identified in the leaf tissue of the four tomato (*Solanum lycopersicum*) control cultivars compared with that of the *R. solanacearum* treated. The plants were incubated for 15 d to allow infection to be established. The heatmap indicates the mean peak intensity of each annotated metabolite following normalization and Pareto scaling of the data. The color scheme is noted in the legend above, indicating fold change increases (red), decreases (green) and significant changes between cultivar conditions (yellow borders). Each row represents a discriminant metabolite feature provided in Table S2, and numbered accordingly. The first four columns show the treated/infected tomato cultivars and the last four indicate the control cultivars.

Extracts prepared from tissues (leaf, stem and roots) of each of the four cultivars were prepared and analyzed on an UHPLC system coupled to a quadrupole time-of-flight high-definition mass spectrometer (qTOF HD-MS) system equipped with an electrospray ionization (ESI) source [27] for the analysis of metabolite classes of different polarities. The data acquisition occurred in both ESI(−/+) modes, which was critical for the analysis of certain metabolite classes (e.g., glycoalkaloids, amino acids and hydroxycinnamic acid (HCA) amides). Visual inspection of the UHPLC-MS base peak intensity (BPI) chromatogram overlays (e.g., Figure 1 for the 1R cultivar; equivalent chromatograms of the 6R, 8S and 9S cultivars are not presented) revealed distinct variations in both the peak intensities (quantitative) along with the presence/absence (qualitative) of peaks across all samples from all tissue types of the tomato cultivars after treatment with *R. solanacearum*. This indicates metabolic variations produced in the tissues of the plant upon pathogen infection. The chromatograms for the ESI(+) mode are shown as Figure S4. Throughout the article, the results obtained from ESI(−) mode were graphically presented in the figures due to the better ionization in this mode (Figure 1, Figure 2, Figure 3 and Figure 4). The study design information, LC-MS raw data, analyses and data processing information, as well as the meta-data have been deposited to the EMBL-EBI MetaboLights database (DOI: 10.1093/nar/gks1004. PubMed PMID: 23109552) with the identifier MTBLS1160. 

### 2.4. Multivariate Data Analyses

Although the chromatographic fingerprints shown (Figure 1 and Figure S4) provide a visual assessment of metabolic reprogramming that occurs upon pathogen treatment between the tissue extracts (leaf, stem and roots) from the four cultivars, informative details can only be achieved by data mining and comparative chemometric analyses to identify more meaningful and underlying structures within the datasets that differentiate the control vs. treated conditions of each cultivar. Due of the large number of chromatograms produced for each of the four cultivars in both ESI modes, all the chromatograms are not presented. The intricate LC-MS data sets from both ionization modes were further subjected to chemometric analysis using explorative along with predictive multivariate statistical tools to reveal *R. solanacearum*-induced feature variation in the metabolomes of the four cultivars. These analyses were also performed to detect signatory metabolites/ bio-markers that were upregulated or synthesized de novo during pathogen infection. Briefly, principal component analysis (PCA) is an unsupervised projection-based statistical tool that allows the exploratory analysis of the data by projecting the original multidimensional dataset on a lower dimensional space, thus permitting the extraction and summarization of underlying group trends in a visual manner, finally displaying the systematic variation present in the data [30,34,35,36]. 

The computed PCA model for all cultivars, controls vs. pathogen treated (Figure 2a) show, without overfitting, clear group clustering of the data from the leaf extracts of the four tomato cultivars. Based on the PCA scores plot data, hierarchical cluster analysis was used to construct a hierarchy of the data which were then projected as a single-linkage dendrogram to represent an outline of the hierarchical data structure of the PCA model [34]. The dendrogram (Figure 2b) illustrates that the data clusters could be separated into two main branches; 9S control (9SC) and 9S treated (9ST) vs. 1R, 6R, 8S, C and T). Further separation is evident in the second branches (1RC, 6RC vs. 8SC, 8ST, 1RT, 6RT), with the final branch subdivided into three groups (1RT, 6RT vs. 8SC vs. 8ST). Lower on the dendrogram, an observation can be made that the control samples show clear separation from that of the corresponding treated counterparts, indicating variation in the metabolite composition of each cultivar. The differential clustering and early merging of the 1R and 6R control branches (Figure 2b) would suggest similar metabolite compositions. The merging of the treatment branches of the resistant sample clusters (1R, 6R) indicates that similar metabolites are produced or have similar relative abundances in response pathogen infection. Interestingly, the proximity of the treated 8S group to that of the resistant cultivar counterparts (1RT, 6RT), illustrates a level of similarity in the metabolite compositions of these cultivars in response to pathogen infection. The distance of the treated 9S branch to the other cultivars suggests that the 9S cultivar activates metabolic pathways and utilizes defense responses different to that of the 1R, 6R and 8S cultivars.

Similarly, comparative PCA models for tissue extracts from leaves, stems and roots from each individual cultivar condition (control and treated) were computed for both ionization modes (Figures S5 and S6 for ESI(−) and ESI(+) modes respectively). The PCA scores plots depicted clear group clustering with distinguishable separation of the control and treated samples Due to the large number of computed multivariate models, the corresponding PCA loadings plots and hierarchical cluster dendrograms are not presented.

The description and use of PCA and orthogonal projection to latent structures-discriminant analysis (OPLS-DA) as multivariate analytical tools, have been thoroughly described in previous literature [26,34,35,37]. As an unsupervised projection-based method aimed at data set dimensionality reduction, PCA is fundamentally a linear-transformation multivariate method, which usually precedes the application of supervised learning tools [36,38]. Moving from the computed PCA models to the application of OPLS-DA as a supervised method, the OPLS-DA scores plots once again provided clear distinction between the control and treated samples for all the conditions mentioned earlier (Figures S7 and S8 for ESI(−) and ESI(+) modes respectively). The OPLS-DA scores plot from the 1R cultivar (Figure 3a) showed a clear group separation between the treated and control samples. The loadings plot (Figure 3b) has a typical S-shape and is used for the identification of the variables which are positively/negatively correlated to the treatment (discriminate variables). The dark triangles indicate features present in both conditions. Relevant variables at the extremes of the loadings S-plot (x,y > 0.05) were selected and represent possible discriminating ions. Variables that contribute to the changes between groups are situated at the upper right - and lower left extremes of the loading S-plot [39,40]. Discriminating ions with a |p(corr)| of ≥ 0.5 and a co-variance value of |(p1)| ≥ 0.5 were selected for metabolite annotation using MS spectral-based metabolite identification [36]. The reliability of the models was evaluated with analysis of variance testing of cross validation (CULTIVAR-ANOVA) as a diagnostic tool, with models of significance having p-values of < 0.05 [30]. The performance of the OPLS-DA models was evaluated using the receiver operating characteristic (ROC) curve (Figure S9c), which consistently remained above the 50% cut-off threshold. This indicates an acceptable discrimination from the OPLS-DA, as a binary classifier, providing adequate sensitivity and specificity [41]. The predictive capacity of the OPLS-DA models was validated using a response permutation test (Figure S9d). The 100 permutated models (*n* = 100) that were generated showed lower R2 and Q2 values compared to that of the calculated OPLS-DA models, indicating statistically reliable computed OPLS-DA models [37]. As previously mentioned, due to the quantity of data produced the OPLS-DA S-plots, ROC curves and permutation plots can be readily accessed by correspondence with the author. The variable importance in projection (VIP) plot (Figure 3c) summarizes the importance of the discriminant variables within the data set and is used as a checkpoint for the selection of statistically relevant ions/variables in the complex data set [42,43]. Blue bars with VIP scores greater than 1 were selected for further analysis and showed a positive correlation to pathogen treatment. 

The VIP scores of the variables are directly proportional to their significance (≥1.00) when comparing variations between two or more group clusters [42]. These ions represent signatory biomarkers distinguishing between control and infected plants. The VIP plot is used as a validation method to avoid the issue of possible bias during variable selection. The endogenous metabolites linked to the treatment were identified using the above-mentioned statistical procedures (OPLS-DA S-plot and the VIP plot) and are summarized (Table S2) with all information linked to metabolite identification. A total of 81 metabolites were detected and shared among the four tomato cultivars. The annotated metabolites are organized into their respective compound classes and numbered based on increasing retention time (Rt). The compounds tentatively identified in the tomato tissues, (Table S2) have been previously described, either within the model organism itself or species within the Solanaceae family. 

### 2.5. Heatmap Visualisation

From the analyzed UHPLC-MS data, eight metabolite classes were clearly identified and annotated which included: amino acids, organic acids, fatty acids, steroidal glycoalkaloids (SGAs), hydroxybenzoic acids (HBAs), HCAs, HCA amides/Phenylamides (PhAs) and flavonoids. The average integrated peak areas of the features were used to construct a heatmap illustrating the relative metabolite concentration differences that occur in the leaf tissues of the cultivars during pathogen infection (Figure 4). The corresponding relative metabolite concentrations for the stem and root tissue can be seen in Figures S10 and S11. Sections in the heatmap that displayed significant variation between the relative metabolite concentrations of the control and treatment conditions were highlighted within yellow borders. 

**Figure 5:**
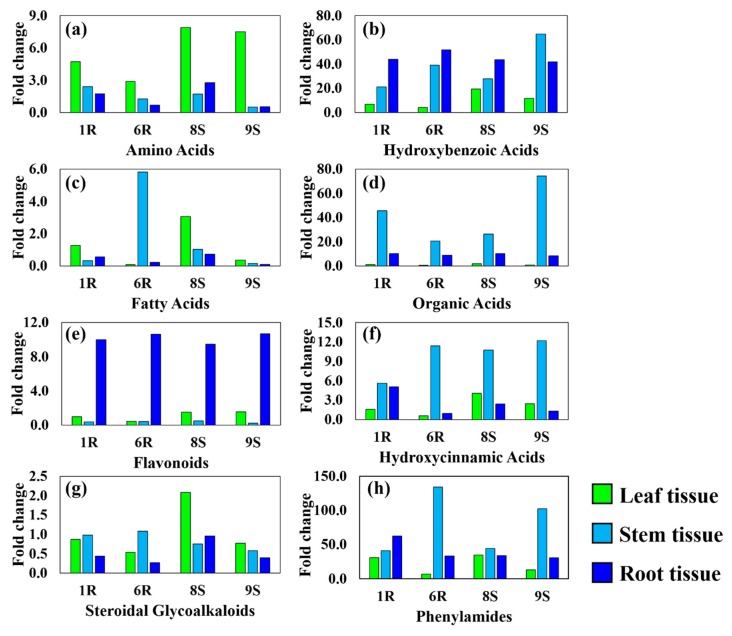
Constructed bar graphs illustrating the respective fold changes of the individual metabolite classes occurring in the leaf (green), stem (cyan) and root (blue) tissues of the four tomato cultivars (1R, 6R, 8S and 9S) after treatment with *R. solanacearum,* 15 d post infection. The respective metabolite classes presented: (**a**) amino acids, (**b**) hydroxybenzoic acids, (**c**) fatty acids, (**d**) organic acids, (**e**) flavonoids, (**f**) hydroxycinnamic acids, (**g**) steroidal glycoalkaloids, (**h**) hydroxycinnamic acid amides/phenylamides. The calculated fold change of each metabolite (Table S2), in the respective plant tissues, was averaged to display the trend of each metabolite class within each tomato cultivar. Due to the high degree of variability observed as a result of fold change variation of each metabolite no error bars were added to the graphs.

The upregulation of the organic acids (Figure 5d) is predominantly observed in the stem tissues, with the 1R and 9S cultivars displaying the highest fold change differences. As intermediates of the citric acid cycle, the synthesis of metabolites such as malic acid (#48), fumaric acid (#49) and (iso)citric acid (#50/#51) may suggest an indirect method of energy production utilized towards combating pathogen infection. The increased fold change differences of isocitric acid, citric acid (free and glycosylated, #52) in the 1R cultivar, citric acid in the 6R cultivar, along with that of fumaric acid in the 9S cultivar are highlighted in Figure 4. The production of ascorbic acid (#53) in the stem would suggest use of the metabolite as a direct antioxidative agent. Similarly, panthothenic acid (#57) was detected as a discriminant metabolite in leaf and stem tissues. The metabolite displayed variable concentrations in the leaf tissues but showed a significant concentration increase in the root tissues of the 8S cultivar, (Figure 4 and Figure S11). 

Three fatty acid compounds were identified as discriminant ions within the datasets. Two isomers of both trihydroxy-octadecadienoic acid (#77, #79) and hydroxy-octadecanedioic acid (#80, #81), along with the presence of 3-amino-13-oxo-tridecanoic acid (#78) were annotated in extracts from the leaf, stem and root tissues, indicated in Table S2. The relative abundance of hydroxy-octadecanedioic acid present within the root tissue, was found to increase among all cultivars during pathogen infection (Figure S11). The trihydroxy-octadecadienoic acid (#77) present in the root tissue showed selective increase abundance only in the 8S cultivar, (Figure S11). The fatty acid metabolites (#78 and #79) displayed a fold decrease in the root tissue of the 1R and 8S cultivars in response to pathogen invasion, while the relative abundance in the 6R and 9S cultivars remained consistent with minor metabolic fluctuations (Figure S11). The trihydroxy-octadecadienoic acid present in the stem tissue of the 6R cultivar displayed a prominent fold change compared to the other cultivar counterparts (Figure S10 and Figure 5c). The observed fold increase in fatty acid metabolites could be the result of lipid signaling in response to infection, precursor synthesis for later lipid peroxyl radical production or membrane destruction accompanied with plant cell death as the result of pathogen presence in the stem [30].

A relatively low fold change of the SGAs among the tissues of the four cultivars, along with a subsequent decrease in the relative abundance in the root tissues could be observed (Figure 5g). This observation highlights the overall distribution of the SGAs throughout all the tissue types of the four cultivars, thus placing emphasis on the classification of SGAs as phytoanticipin compounds in tomato. The nitrogen-containing steroids α-tomatine (#74, #75) and dehydrotomatine (#72), are the two hallmark SGAs typically found in tomato. In conjunction with the overall view provided in Figure 5g, a decrease in the relative SGA abundance can be observed in the stem tissue of the 9S cultivar (Figure S10). Other SGAs derivatives identified (Table S2) include: hydroxytomatine (#71), lycoperoside A/B/C (#73) and a glycosylated form of the tomatidene core moiety, tomatidene dihexoside dipentoside (#76). All the identified SGAs with a recorded presence in root tissue (Table S2, excluding lycoperoside) demonstrated a significant decrease in roots of the 6R cultivar (Figure S11). As *R. solanacearum* is a soil-borne pathogen that gains entry through the root tissue, the decrease in SGAs can be suggestive to a functional role of fending off or retarding pathogen progression.

In the HBA class, the metabolites identified included salicylic acid (SA—2-hydroxybenzoic acid) and its conjugates (#63, #65, #67). SA, free (#63) and glycosylated (#67), were only detected as discriminant ions in the leaf and root tissues (Table S2). Interestingly, the glycosylated derivative of methyl salicylate (MeSA, #65) was distributed throughout the leaf, stem and root tissues (Table S2), and subsequently also displayed an increase in relative metabolite abundance during pathogen treatment among all cultivars (Figure 4, Figures S10 and S11). The four diHBAs (#61, #62, #68, #69) detected were annotated as glycosylated forms (either as hexose or pentose—Table S2). The first two (#61, #62) diHBAs were found in all tissue types, while the latter two (#68, #69) were only detected in the leaf tissues of all cultivars Interestingly, the benzyl alcohol found (#64) showed a significant decrease in concentration in the leaf tissues of the 1R and 6R cultivars upon pathogen treatment, while the leaf tissues of 8S and 9S displayed less prominent fluctuations in metabolite concentration (Figure 4). Variable cultivar-related concentrations were observed in the stem and root tissue (Figures S10 and S11). In addition to induced synthesis due to pathogen infection, the benzoic acids/alcohols and glycosylated derivatives, similarly to the HCAs, may also be utilized as a storage pool of resources during normal plant homeostasis, that can be redirected for the rapid synthesis of defense-related compounds during perturbations [44]. The putatively identified metabolite, 3-methylbutyl 6-apio-furanosyl-glucopyranoside (#66), was detected in all tissue types of the tomato cultivars but demonstrated an interesting trend among the 1R and 9S cultivars, [45]. The metabolite exhibited an increase in the leaves of the 9S cultivar while decreasing in the 1R cultivar, (Figure 4). The stem tissue pointed to variable cultivar-related variation that could not be explained (Figure S10), while the root tissues of the 1R and 9S cultivars both showed an increase (Figure S11). The observed variability in concentration among the tissue types points to cultivar variation in the speed and manner of resource redistribution between the host tissues. 

The presence of glycosyl conjugates of the HCAs, caffeic acid (#1, #2, #7), ferulic acid (#6, #13) and sinapic acid (#9, #10, #14), identified at the sites of initial and established infection, e.g., the root and stem tissue (Figures S10 and S11 and Table S2), represent special significance as secondary metabolites that may serve as a metabolic pool of derivatized HCAs that can readily be injected (upon hydrolysis) into the phenylpropanoid pathway for the rapid production of phytoalexins, or incorporated into the plant cell wall as a strategy of halting pathogen progression [30,46]. Dihydrocaffeic acid (#19—glycosylated) was only detected in the leaf tissues and showed a remarked decrease in concentrations in the treated samples. The conjugation of both caffeic acid (#3, #4) and ferulic acid (#18) with glucaric acid was detected in both the leaf and root tissues of all cultivars, with the synthesis of caffeoyl glucaric acid isomers upregulated in the leaf tissues (Figure 4). As described before, conjugates of the HCAs such as quinic acid esters, may serve as a metabolic pool that can be used to enhance metabolic flow through the phenylpropanoid pathway. The *cis*/*trans* forms of the 3-caffeoylquinic acids (#5, #8—CQA) and 5-caffeoylquinic acids (#12, #16), along with the *trans* form of the 4-caffeoylquinic acid (#15) were shown to accumulate in all the tissue types of all four cultivars. The *cis* form of the 4-CQAs could not be accurately detected and characterized. The *trans*-5-CQA was detected in the tissues of all the cultivars (Table S2) as well as a subsequent increased synthesis of the metabolite in the 1R cultivar (Figure 4, Figures S10 and S11). The *cis*-5-CQA was characterized with a substantial increase in the leaf and root tissues of the 1R and 6R cultivars (Figure 4 and Figure S11). The *cis*-forms of the HCAs in Solanaceae have been reported to play a role in plant defense [47]. Similarly, an increased synthesis of the *trans*-4-CQA was detected in the leaf tissue of the 1R cultivar (Figure 4). Both the *trans*-3-CQA and *trans*-5-CQA exhibited similar responses, showing an increase of the metabolites in all tissue types of all cultivars. The *cis*/*trans* forms of the 5-feruloylquinic acids (#11, #20—FQA) were found in all tissues of all cultivars, while both *cis*/*trans* isomers of the 5-coumaroylquinic acids (#17, #23—CoQA) were present in the leaf tissue, with only the *cis*-5-CoQA subsequently found in the stem tissue of all four cultivars, (Table S2). Additionally, a glycosylated form of the FQA metabolite was detected in the root tissues, demonstrating a positive correlation between metabolite concentration and pathogen exposure (Figure S11). With the 4-hydroxycoumarin (#22, #24—glycosylated) secondary metabolite isomers, a marked increase in concentrations in the leaf and root tissues were observed in the 1R and 6R cultivars, (Figure 4 and Figure S11). Interestingly, a mixed ferulic acid: sinapic acid conjugate was identified in the leaf tissues, putatively identified as feruloyl sinapoyl glucaric acid (#25) which exhibited a marked increase in the 6R and 8S cultivars.

The PhA metabolite class displayed the highest degree of metabolic synthesis in the leaf tissues of the tomato cultivar in response to *R. solanacearum* treatment (Figure 4). Similar patterns could subsequently also be observed in the corresponding stem and root tissues (Figures S10 and S11). The heatmap shows that secondary metabolites like feruloyl tyramine (#35, #36, #38—glycosyl and methoxy-conjugates), caffeoyl putrescine (#26, #29—glycosyl conjugate) and coumaroyl tyramine (#32, #33—glycosyl conjugate) were predominant compounds in the PhA class, accumulating in all plant tissues (Figure 4 and Table S2). The synthesis of the polyamine putrescine was found to be upregulated in response to pathogen infection, with the detection of the molecule occurring as cinnamic acid conjugates (#27—feruloyl putrescine and #31 sinapoyl putrescine). The presence of the putrescine derivatives has been described as associated with an oxidative environment or drought stress [48,49,50]. In addition to the detection of the documented metabolites, other PhAs, such as coumaroyl- (#34) and feruloyl dopamine (#37) derivatives, as well feruloyl serotonin isomers (#28, #30) were found to be induced in the infected tissues. The annotated isomers of feruloyl serotonin were found to be distributed throughout the tissues of the tomato cultivars. The isomers occurred in the greatest abundance in the leaf tissue of the 6R cultivar (Figure 4) and a comparative relative abundance among the root tissue of the 1R and 6R cultivars (Figure S11). The *trans*-feruloyl serotonin was present in equal abundance in the stem tissue of all tomato cultivars, while the presence of the *cis*-form was selectively upregulated within the 8S cultivar (Figure S10). The presence of these compounds is supported by and in agreement with previous literature [51,52,53,54,55]. Diferuloyl spermidine (#39) was detected in the leaf and root tissues of the four cultivars. The compound showed a decrease in concentration in the leaf tissue of the 8S cultivar (Figure 4), while a subsequent increase in the root tissue was observed (Figure S11). The rapid synthesis and high fold changes of these metabolites in the tissues of the tomato cultivars during the infection process would suggest a functional role of the PhAs in plant defense. Accumulation of PhAs after treatment with *Pseudomonas syringae pv. tomato* has been linked to enhanced resistance of the host plant, tomato [51]. The high fold changes found for the PhAs in the leaf, stem and root tissues upon bacterial treatment is the result of the de novo synthesis of these PhAs, indicating a phytoalexin role for these compounds in tomato.

The fold change of each metabolite belonging to one of the respective classes: the amino acids, organic acids, fatty acids, SGAs, HBAs, HCAs, PhAs and flavonoids, was averaged for each of the tissue types and presented as bar graphs (Figure 5). Figure 5 shows the pooled fold changes of each metabolite class providing a relative holistic view of the metabolic reprogramming that occurs in the tissues of the tomato cultivars during infection with *R. solanacearum*. Figure 5a gives an indication that the synthesis of amino acids and derivatives, e.g., glutamic acid (#54), phenylalanine (#56) and acetyl tryptophan (#59), are significantly upregulated in the leaf tissue of all cultivars, while a significant reduction in synthesis is observed within the stem and root tissues (Figures S10 and S11). The putatively identified metabolite acetyl leucine/isoleucine (#58) was absent in the stem tissue but displayed a significant increase in concentration in both the leaf and root tissues of all tomato cultivars, grouping close to that of the PhAs in Figure 4 and Figure S11. Hydroxytryptophan (#60), an intermediate in the synthesis of serotonin, demonstrated a constant abundance (1R, 6R and 8S) or significant decrease in concentration (9S) in the leaf tissue of the cultivars, (Figure 4). A marked increase was only observed in the stem of the 6R cultivar and a decrease in relative abundance was noted in the roots of all cultivars, (Figure S11). Unexplained cultivar-related metabolic shifts were observed in the leaf, stem and root tissues for the glutamic acid derivative, pyroglutamic acid (#55), reported to enhance antioxidant defenses. 

The upregulation of the HBA class (Figure 5b and Table S2) can be observed in the root and stem tissues of all the cultivars. The overlap between the sites of HBA upregulation with that of the established infection sites of the pathogen can indicate a direct functional role in plant defense (either as signaling molecules, constituents used in cell wall reinforcement or antimicrobial compounds). 

**Figure 6:**
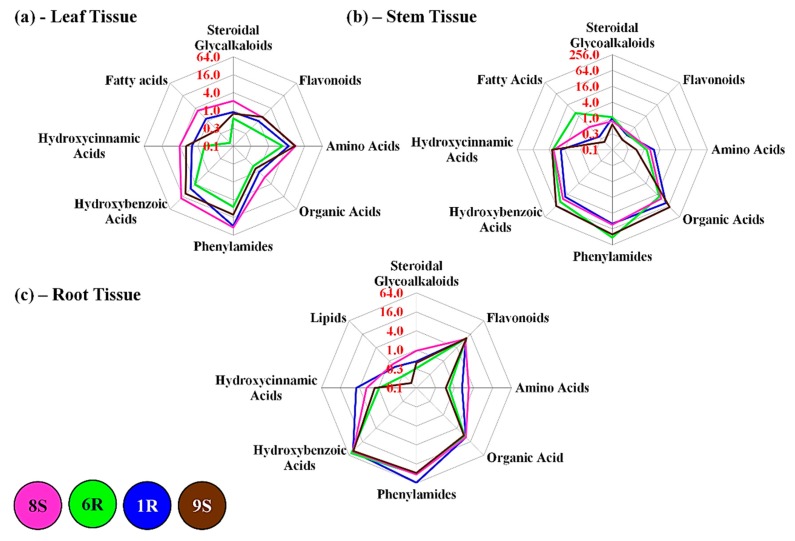
Representative radar charts showing the comparative fold changes of the main metabolite classes present in the (**a**) leaf, (**b**) stem, (**c**) root tissue of the four tomato cultivars (1R, 6R 8S and 9S) after treatment with *R. solanacearum,* 15 d post infection. The generated fold change datasets were averaged and illustrated as log2- transformed values.

Figure 5h shows the overall accumulation of the PhA class in the different tissue types. The highest degree of metabolite abundance was observed in the stem tissue of the 6R and 9S cultivars, in contrast to the 1R and 8S cultivars that showed a similar accumulation in both the stem and root tissues (Figure 5h). The accumulation of amide conjugates containing putrescine and tyramine upon infection with the pathogen indicate that the metabolites could be used as candidate biomarkers. The averaged fold change differences of each metabolite class were combined to produce radar charts (Figure 6), comparatively displaying the metabolomes of each plant tissue (leaf, stem and root) along with the metabolic variations between the tissue types of the tomato cultivars. However, due the large variations observed in each metabolite class, partially the result of de novo phytoalexin synthesis, the metabolic reprogramming that occurs in the pathway of one class may be overshadowed by another.

Figure 6a shows the comparative leaf metabolomes of the four cultivars, with the 1R and 6R showing the lowest abundance of metabolites from each respective class. The 8S cultivar visually shows the highest concentrations of metabolites in the leaf tissue. Metabolite classes with the highest fold changes in the leaf tissue included the amino acids and PhAs. Figure 6b shows the comparative stem metabolomes of the four cultivars. A described before, the metabolite classes with the highest fold change differences in the stem tissue were shown to be the PhAs, HCAs, HBAs and organic acids. One clear observation is the increased fatty acid class concentration in the 6R cultivar stem tissue that could also be observed in Figure 5c. The comparative root metabolomes of the four cultivars are shown in Figure 6c. The metabolite classes that demonstrated high fold changes were flavonoids, HBAs and PhAs, shown in both Figs. 5 and 6c. The high accumulation of the PhAs and HBAs could be observed throughout the tissue metabolomes of the tomato cultivars Figure 6 also shows that the metabolomes of the three tissue types comparatively differed from each other, along with additional inter-cultivar variation. 

The high fold changes observed for the flavonoids in the root tissues (Figure 5e, Figure S11 and Table S2) can be attributed by the presence of rutin (#43), eriodictyol (#44—glycosylated) and kaempferol (#47—glycosylated). The presence of the flavonoids concentrating in the root tissues can be the result of pathogen presence, indicating a participating role of flavonoids in plant defense. Quercetin and associated glycosylated derivatives (#40, #41, #42, #45) were found in the leaf tissues. The remaining abundance of flavonoids in the leaves may assist as antioxidants against the drought-like environment experienced in the leaves of the plant once water movement through the stem is blocked due to the established infection. Previous literature has described the inherent antimicrobial activity of the flavonoid structure, along with the multiple hydroxyl groups conjugated to the core moiety which bestows a pathogen-derived free radical neutralization potential [30]. The abundant presence of the HCAs in the stem tissue (Figure 5f, Figure S10 and Table S2) of all cultivars, support the functional role of this metabolite class in plant defense. All cultivars produced a >3-fold difference in HCAs presence in stem tissues upon pathogen infection. The 1R cultivar demonstrated the lowest concentration of HCAs among the cultivars but produced the highest fold change in the root tissue, suggesting a ratio of resource distribution among the infected tissue sites (stem and roots). 

## 3. Discussion

Through a highly advanced innate immune system, plants have evolved an adaptable metabolic capability to detect potential threats and deploy a wide range of resistance mechanisms [56,57]. These mechanisms include the synthesis and deployment for various chemical compounds to ward off attempted infection and involves the reorganization of the metabolome to produce secondary metabolites in support of defense. The production of specific secondary metabolites can elevate the ability of plants to survive and overcome local environmental challenges [58]. Our non-targeted metabolomics results have highlighted dynamic changes in the metabolomes of infected vs. uninfected tomato plants, adaptations made in an attempt to defend against infection by *R. solanacearum*. The metabolite classes identified include: amino acids, organic acids, fatty acids, SGAs, HBAs, HCAs, PhAs and flavonoids (Table S2 and Figure 6). Our previous study described the occurrence of these metabolite classes in the four cultivars, as well as the defense-related roles of the pre-existing SGAs, flavonoids and fatty acids [30]. This discussion focuses on the significance of HCAs, HBAs, and PhAs as inducible metabolites in response to *R. solanacearum* treatment. The relevant sub-branches of the phenylpropanoid pathway can be viewed at the Kyoto encyclopedia of genes and genomes (KEGG) pathway database.

### 3.1. Hydroxybenzoic Acids

HBAs are C6–C1 aromatic carboxylic acids that serve as precursors for a wide array of essential secondary metabolites participating in crucial roles in plant fitness [44]. The best studied plant HBA is SA which, along with its derivatives, is considered one of the key endogenous plant hormones utilized in cell signaling in response to biotic and abiotic perturbations [59,60]. Several diHBAs (e.g., gentisic acid and protocatechuic acid) have been implicated in plant defense, either acting as antimicrobial compounds or as additional components of the SA signaling system for the activation of inducible defenses [61,62]. A study performed on the modulation of SA-mediated plant immune responses suggested the presence of a positive feedback loop between diHBAs and SA synthase used to regulate SA concentrations [63]. Accumulation of SA triggers the oxidative burst and ultimately leads to programmed cell death (PCD/the hypersensitive response, HR) and increased plant resistance to pathogens through systemic acquired resistance (SAR) [63].

### 3.2. Hydroxycinnamic Acids

The HCAs have been reported as defense-related compounds in plants. Derived from the shikimate and phenylpropanoid pathways, HCAs, together with their derivatives, have been shown to act as direct antimicrobial-, antioxidant- and cell wall reinforcing compounds [64,65,66]. Strains of *R. solanacearum* have the ability to degrade HCAs, a trait that contributes to pathogen virulence by protecting the pathogen from HCA toxicity, thereby facilitating root entry [67]. HCA degradation is described as a broadly conserved trait within the *R. solanacearum* species complex (RSSC), where strains lacking the trait demonstrates a reduction in virulence accompanied by an increased susceptibility to HCA toxicity [67]. HCAs play functional roles in plant defense by direct incorporation into the cell wall cross-linking polymers, structurally reinforcing the physical barriers; additionally also serving as precursor monomers utilized in lignin formation [67,68]. The discovery of hydroxycoumarins in the treatment dataset indicates a possible role as antimicrobial compounds. Recent evidence has also showed the susceptibility of *Ralstonia* spp. to hydroxycoumarins [69]. Plants also contain phenolic-containing cells lined within the vascular systems that release the phenolic contents into the xylem-lumen upon pathogen infection [70].

### 3.3. Phenylamides

Polyamines are aliphatic organic compounds containing multiple amino groups within the core structure [71]. Phenylamides (Table S2) are produced by a conjugation reaction between polyamines and HCAs [72]. Literature has supported the idea that the synthesis of these secondary metabolites is induced by a variety of abiotic perturbations [73,74,75]. In addition to the above-mentioned, phenylamides have been shown to accumulate during interactions between the host plant and pathogens [76]. Upon pathogen infection, phenylamides display great metabolic plasticity, being able to contribute and enhance plant defense by participating in a variety of induced mechanisms. Firstly, restriction of pathogen development due to both antioxidant and antimicrobial properties [66,72,77] associated with compounds such as feruloyl tyramine, feruloyl dopamine and feruloyl methoxytyramine [78]. Anti-bacterial and antioxidant activities of leaf extracts prepared from various Solanaceae species with special focus on the PhAs and the steroidal glycoalkaloids metabolite classes have also been reported [79]. The PhAs possessed good activity against the pathogens tested but results suggested that the phenolics may not be the sole contributor, i.e. the glycoalkaloids, to the antioxidant capacity of the plants tested. Another study compared the antioxidant activity of PhAs with that of the HCAs, using free hydroxycinnamic acids (e.g., coumaric-, caffeic-, ferulic- and sinapic acid) as the positive controls. Results suggested that the PhAs exhibited a higher radical scavenging capacity compared to the corresponding HCAs but, lower compared to the free forms [80]. Secondly, PhAs can undergo oxidative polymerization for the direct incorporation into the cell wall, forming intermolecular bridge structures that maintain the structural integrity of the barrier by strengthening linkages between the various cell wall components, thereby making the barrier more resistant to mechanical, chemical and enzymatic breakdown [72]. Thirdly, through an enzyme mediated reverse reaction, polyamine (PAO)- and diamine oxidases (DAO), HCA-amides can be broken down to polyamines resulting in the release of hydrogen peroxide (H_2_O_2_) as a degradation product of the reaction [81,82]. The production of H_2_O_2_, and other free radical species, restricts pathogen development by strengthening the cell wall through oxidative cross-linking, by directly attacking and disrupting the pathogen, and by mediating the initiation of the HR. The reaction is initiated by recognition of pathogen attack, closely followed by an oxidative burst, the induction of defense-related gene expression and finally hypersensitive cell death [82]. This form of PCD is commonly utilized in plants to kill off compromised cells, thereby protecting healthy cells and limiting the development of the pathogen at the site of attempted invasion. Lastly, it has been proposed that polyamines such as putrescine play a role in regulating stomatal closure through the reduction of the stomatal aperture [83]. PhA-regulation of stomatal movements involves maintaining inward potassium channels within guard cells [73]. Since *R. solanacearum* gains entry to the host through the root system and establishes itself in the xylem channel to essentially block the distribution of water to the upper portion of the plant, it can be hypothesized that the host experiences a simulated drought environment leading to the rapid synthesis of both PhAs and abscisic acid (ABA). Biosynthesis of the hormone occurs in response to water deficiency and ABA is commonly associated with abiotic stress by reducing water loss through stomatal pore closure [83,84]. Controversy still surrounds the idea whether ABA modulates polyamine synthesis at a transcriptional level or vice versa in support of a functional role in water loss prevention [73,85]. 

An overall evaluation reveals that all the tomato cultivars responded in similar fashion in response to pathogen infection (Figure 5b,e) with similar levels of HBAs in the stem tissue and flavonoids accumulating in the root tissues. All four cultivars displayed traits of tolerance towards the pathogen once examined from various perspectives such as: the abundance of precursors that can be readily used, the decreased concentration of defense metabolites due to utilization in plant defense, as well as the speed and timing of secondary metabolite production. Using a method of regression from the phenotypic state it could be suggested that the 6R cultivar shows the best ability in fending off the pathogen due to the highest abundance of defense-related metabolites (the HBAs, HCAs, PhAs and flavonoids) in the stem and root tissues (Figure 6b,c), the known sites of pathogen entry and establishment. The decreased concentrations of secondary metabolites in the leaf tissue (Figure 6a) could be explained by the possible transport to the sites combating infection. Although all four cultivars displayed pronounced symptoms indicative of *R. solanacearum* infection and were past the point of possible recovery, the metabolic profiles suggested a good defense response launched by cultivars to protect against infection. Bacterial wilt is generally a slow developing disease; but the subsequent concentration of inoculum, along with the direct method of inoculation in the roots and stems rapidly accelerated pathogen development and the infection process. The combination of soil drenching, root cutting, and direct stem inoculation facilitated pathogen entry and establishment in the host plants. 

## 4. Materials and Methods 

### 4.1. Plant Cultivation

The seeds from four tomato cultivars, STAR9001 (1R), STAR9006 (6R), STAR9008 (8S) and STAR9009 (9S) were obtained from a tomato breeding program (Stark Ayres, Pty. Ltd. Bredell, South Africa) and cultivated in 10 cm pots containing germination soil mixture (Culterra, Muldersdrift, South Africa). Each cultivar was grown in triplicate under controlled greenhouse conditions: a light/dark cycle of 12 h/12 h, the light intensity set at 60 µmol m^−2^s^−1^ and the temperature regulated to between 22–24 °C. 

### 4.2. Pathogen Inoculation

A highly virulent *R. solanacearum* BD 261 strain (Coutinho collection, University of Pretoria, RSA, GenBANK Accession number KY 709230, approved on July 3, 2017), from South African origin belonging to race 2, biovar 3, was used for inoculation of the tomato cultivars. The bacteria were grown in Luria Bertani liquid media for 24 h at 28 °C. Once plant maturity was reached (8 weeks) the cultivars were inoculated by drenching the soil with 500 mL of an OD_600_ = 0.06 inoculum suspension of *R. solanacearum* (corresponding to approximately 10^8^ cfu/mL^−1^). Sterile hypodermic needles and syringes were used to also administer a droplet (2 μL) of the inoculum or sterile distilled water (control) into the vascular tissue of each plant stem [33,86]. The roots visible at the top of the pot were also cut to allow a point of pathogen entry. The plants were incubated for 15 d to allow infection to be established (Figure S2). The whole stem (>35 cm), the whole of the root system and leaves 3^rd^ and above from the base were harvested, instantly frozen in liquid nitrogen to quench metabolic activity, and stored in –80 °C until metabolite extraction.

### 4.3. Bacterial Re-Isolation and Pathogenicity Trials

*R. solanacearum* was isolated from the midstems of randomly selected treated plants post-harvest. The midstems were treated with 70% alcohol for 10 s rinsed, then allowed to dry in a laminar flow for 10 min. The stem tissue was homogenized in sterile demineralized water. The homogenized solution was passed through a sieve to remove bulky plant material. One mL of the solution was plated with two replicates on both triphenyl tetrazolium chloride (TTC) medium and selective South Africa-Elphinstone (SMSA-E) medium [33,87] and incubated at 28 °C for 48 h. The colonies had a mucoid appearance and were white with a red-pink center on the TTC- and SMSA-E media plates. Subsequently, a tomato cultivar (Stark Ayres), was infected with the isolated strain to prove Koch’s postulates and validate the first results.

### 4.4. Symptom Index

The disease severity or wilt index was calculated over 15 d of inoculation using the following formula: Disease index (%) = [∑ (ni × vi)/(V × N)] ×100, where the ni = number of plants with the respective disease rating; vi = disease rating; V = the highest disease rating (5); and N = the number of plants observed [33]. The development of disease was evaluated using index 0–5 [5]. A score of 0 indicated no leaves wilted and scores of 1–5 indicated 25%, 26–50%, 51–75%, 76–90% and 91–100% wilting respectively. 

### 4.5. Metabolite Extraction and Sample Preparation

Tissues frozen with liquid nitrogen were pulverized with a mortar and pestle. Two grams of leaf and stem material and 1 g of root material from each cultivar were extracted with 80% methanol in a 1:10 (*w*/*v*) ratio. The samples were sonicated twice in a sonicator bath for 30 min at 20 °C. Cell debris was pelleted with a bench top swinging-bucket centrifuge set at 5525× *g* and 5 °C for 20 min. The supernatants were evaporated to 1 mL using a rotary evaporator at 55 °C, carefully transferred into 2 mL microcentrifuge tubes and dried in a heating block overnight at 55 °C. The samples were then reconstituted in 500 µL of 50 % HPLC-grade Methanol: MilliQ water solvent. The samples were filtered through 0.22 µm nylon syringe filters into chromatographic vials, capped and stored at 4 °C until analyzed. Three biological repeats for each sample were prepared and analyzed in triplicate (technical repeats) to gain accuracy and precision (*n* = 9). The overall results generated were representative of two independent experiments (*n* = 18). A quality control (QC) sample consisting of aliquots from all the samples was also prepared to monitor the stability of the samples, the instrumentation and analyses.

### 4.6. Ultra-High Performance Liquid Chromatography-Mass Spectrometry Analyses

Two μL of each sample extract was analyzed on an UHPLC (Waters Corporation, Manchester, UK). The analytes were separated on an Acquity HSS T3 reverse-phase column (2.1 mm × 150 mm × 1.7 µm; Waters Corporation, Milford, MA, USA) using a binary solvent system consisting of acetonitrile (Romil Chemistry, Cambridge, UK): MilliQ water, with both solvents containing 0.1% formic acid (FA, Sigma, Munich, Germany). A gradient elution method was used over a 30 min run with a flow rate set to 0.4 mL min^−1^. The elution was started at 2% (*v*/*v*) acetonitrile from 0–1 min, raised to 60% acetonitrile from 1–22 min, taken up to 95% from 22–23 min then kept constant at 95% acetonitrile from 23–26 min. The composition of the mobile phase was then reverted to 2% acetonitrile from 26–27 min, for column cleaning and equilibration from 27–30 min [28]. 

### 4.7. Quadrupole Time-Of-Flight Mass Spectrometry (q-TOF-MS) Analyses

The chromatographically separated metabolites were detected with the aid of a quadrupole time-of-flight high-definition mass spectrometer (MS) detector (qTOF HD-MS) system equipped with an electrospray ionization (ESI) source (Synapt G1 high definition mass spectrometer, Waters Corporation) set to acquire data in both positive and negative ionization modes. The MS conditions were as follows: capillary voltage of 2.5 kV, sample cone voltage of 30 V, microchannel plate detector voltage of 1600 V, desolvation temperature of 450 °C, source temperature of 120 °C, cone gas flow of 50 L·h^−1^, desolvation gas flow of 550 L·h^−1^, *m*/*z* range of 50–1500, scan time of 0.2 s, interscan delay of 0.02 s, mode set as centroid, lockmass flow rate of 0.1 mL·min^−1^, lockmass set as leucine enkephalin (554.2615 Da) and mass accuracy window of 0.5 Da. High purity Helium was used as desolvation-, cone- and collision gas. The MS analyses were set to perform unfragmented as well as four fragmenting experiments (MS^E^) simultaneously by collision energy (CE) ramping from 10 to 50 eV. Data acquisition at these various CEs was performed to facilitate metabolite fragmentation for later assistance in downstream structure elucidation and compound annotation [28]. 

### 4.8. Data Analyses

The UHPLC-ESI-MS data sets were analyzed with Markerlynx XSTM software (Waters Corporation), with the addition of other statistical programs for multivariate data analysis. The raw UHPLC-ESI-MS data was processed with MarkerLynx XSTM 4.1 software, with the following parameters: 0.60–21 min Rt range of the chromatograms an *m/z* mass range of 50–1500 Da. The Rts were allowed to differ by ± 0.20 min and the *m/z* values by ±0.05 Da. The mass tolerance was 0.01 Da and the intensity threshold was 10 counts. Only the data matrices with noise level less than 50% (MarkerLynx cut-off) were retained for downstream data analyses. The MarkerLynx application uses the patented ApexPeakTrack algorithm to perform accurate peak detection and alignment. Furthermore, MarkerLynx performs sample normalization, based on total ion intensities of each defined peak. Prior to calculating intensities, the software performs a modified Savitzky-Golay smoothing and integration [24,39,88]. The generated data matrices were exported into Soft Independent Modeling of Class Analogy (SIMCA) software, version 14.0 software (Umetrics, Umea, Sweden) for multivariate statistical analyses. To put all variables on equal footing, and adjusting for measurement errors, the data was Pareto-scaled prior to chemometric modeling. A nonlinear iterative partial least squares algorithm (in-built within SIMCA software) was used to handle missing values, with a correction factor of 3.0 and a default threshold of 50 %. Two unsupervised methods, PCA and HCA, as well as a supervised method, OPLS-DA, were employed. The OPLS-DA models were used to compare the control and treated samples for each cultivar, for the identification of *m*/*z* ions responsible for the discrimination between the two groups [26]. A seven-fold cross-validation (CV) method was applied as a tuning procedure in computing the models [39]. Thorough model validation steps were consistently applied, and only statistically valid models were examined and used in data mining for metabolite annotation.

### 4.9. Metabolite Annotation and Qualitative Comparison

Chemical- and structural identities of the metabolites were elucidated using their respective mass spectral patterns obtained during the MS analysis. MS spectral-based metabolite identification was performed based on sufficient and accurate mass fragment information, accurate calculation of the elemental composition of each *m*/*z* feature and database searches for possible metabolite annotation. MassFragment, a built in Markerlynx XSTM software tool, was utilized for assigning possible structures to observed fragment ions of the precursor metabolite features using novel algorithms. The putative empirical formula of each statistically significant extracted ion peak (XIC) in the mass spectra was obtained and searched in databases: ChemSpider, Dictionary of Natural Products, PubChem, Metlin, the Tomato Metabolome database (MoTo), KEGG Compound database, for the identification of possible compound matches [30,45,46,89,90]. Metabolites were tentatively identified/annotated to level 2 of the Metabolomics Standard Initiative (MSI) [91,92].

### 4.10. Relative Quantification

Upon identification of statistically significant biomarkers, the relative concentrations of the features were calculated for the control and treated samples in the four cultivars, as represented by peak intensities obtained from the original chromatograms. The fold changes of the features were calculated and imported into the web-based tool suite Metaboanalyst, for further statistical analysis. This included the construction of heatmaps as graphical representations showing the relative peak areas of the statistically selected biomarkers in the treated tissues of the four cultivars allowing for functional interpretation of the data structure [90]. To visualize the magnitude of change of multiple common variables in one chart, radar plots were constructed based the averages of the generated fold change datasets and illustrated as log2-transformed values. 

## 5. Conclusions

Untargeted metabolomics detected metabolites in tomato that could be used as candidate biomarkers associated with *R. solanacearum* infection. The 81 secondary metabolites identified by multivariate data analysis originated from both primary and secondary metabolism. The metabolite classes identified to be predominantly associated with the treatment included the HBAs, HCAs, PhAs, and flavonoids, known to play integrative roles associated with plant defense mechanisms. The relative abundance of pre-existing secondary metabolites (fatty acids, organic acids, amino acids, SGAs, etc.) is linked to the capacity of each cultivar to preferentially utilize these resources (i.e., phytoanticipins) in basal resistance strategies, or for the synthesis of specialized antimicrobial compounds (i.e., phytoalexins) that participate in the induced defense response to resist pathogen infection. The elevated levels of HBA-derived compounds, e.g., dihydroxybenzoic acids and salicylic acid, after pathogen infection is consistent with their functional role as pathogen-induced signals, activating plant defenses. The HCAs and flavonoid metabolite classes, e.g., 3-CQA, 5-FQA, rutin, etc., serve an important role in plant defense; either as cell wall precursors, radical scavenging molecules or direct antimicrobial compounds. Several phenylpropanoid derived compounds belonging to the PhA metabolite class, such as feruloyl tyramine, feruloyl methoxytyramine and caffeoyl putrescine, were found to accumulate within the tissues of the tomato cultivars and demonstrated a positive correlation to the pathogen treatment. The PhA metabolites mentioned can be described as phytoalexins of tomato due to the rapid accumulation in the plant tissues after *R. solanacearum* treatment and can subsequently also be utilized as further candidate biomarkers indicative of perturbations associated with biotic stress. The metabolomic profiling generated new knowledge regarding the underlying physiological strategies employed by *Solanum lycopersicum* to fend off *R. solanacearum* attack and contribute to ongoing investigations to understand molecular mechanisms underlying plant responses to pathogen attack and infection.

## Supplementary Materials

The following are available online at https://www.mdpi.com/1422-0067/20/16/3945/s1.
