# Supplementary material for: Metabolomic Profiling of the Host Response of Tomato (Solanum lycopersicum) Following Infection by Ralstonia solanacearum
Source: Int J Mol Sci. 2019 Aug 14;20(16):3945. doi: 10.3390/ijms20163945 (PMC6720392; doi:10.3390/ijms20163945)
Supplement: Supplementary file 1 [file ijms-20-03945-s001.pdf]

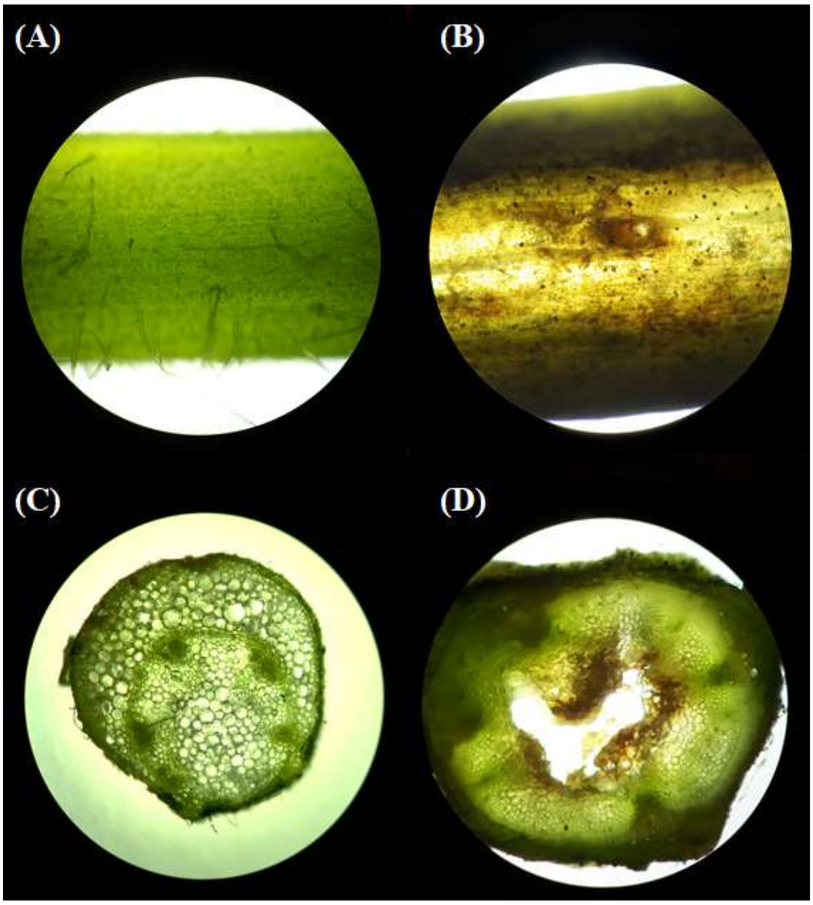

Figure S1. Lower stem symptoms of tomato infected with *Ralstonia solanacearum* view under a light microscope at 10X magnification. **(A)** A longitudinal stem section of the 8S cultivar, and **(B)**, the brown discoloured longitudinal section stem of the infected 8S cultivar. A cross section cut through the stems of the **(C)** control and **(D)** the infected 8S cultivar.

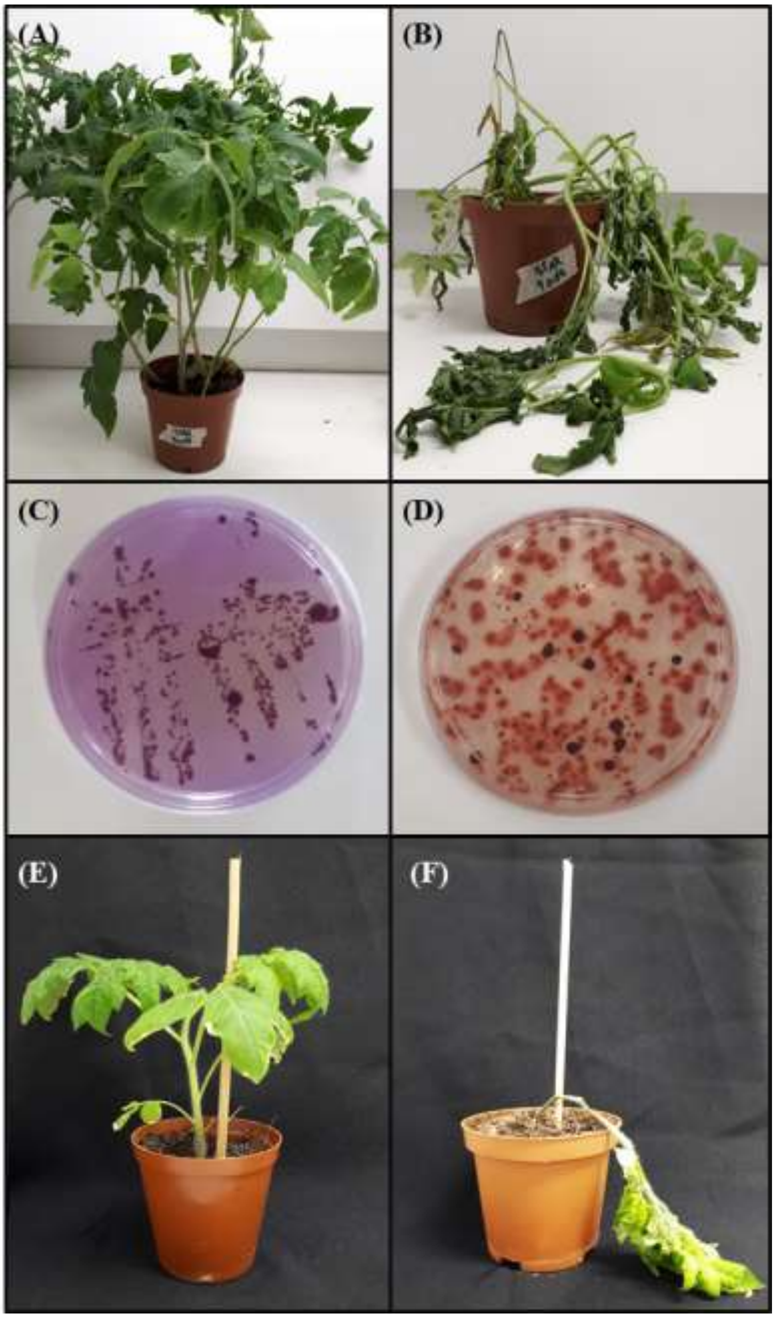

Figure S2. The morphological symptoms presented by tomato plants after infection with *Ralstonia solanacearum*. **(A)** The mature untreated 8S cultivar. **(B)** An infected 8S cultivar. **(C)** Colonies of *R. solanacearum* isolated from the stem of an infected 8S cv, plated on SMSA-media. **(D)** A single colony isolated from the SMSA-media culture that was re-plated onto TTC-media. Avirulent colonies are identified as having a dark red colour while virulent colonies present with a white-pink colour. The strain shows a mucoid or fluidal morphology, indicating virulence. **(E)** An untreated "Moneymaker" tomato cv. **(F)** A "Moneymaker" cv. with known susceptibility to *R. solanacearum*, treated with the strain isolated from the SMSA-media.

Table S1. Information guide of tomato (*Solanum lycopersicum*) cultivars used in this study, indicating high/intermediate resistance to a variety of viral, bacterial, fungal and nematode pathogens.

Disease Resistance Phenotypes				
	**STAR 9001 (1R)**	**STAR 9006 (6R)**	**STAR 9008 (8S)**	**STAR 9009 (9S)**
High Resistance	Va:1 / Vd:1<br>Fol:1-2 / **Rs**	Va:1 / Vd:1<br>Fol:1-2 / **Rs**	Va:1 / Vd:1<br>Fol:1-2	Va:1 / Vd:1<br>Fol:1-2
Intermediate resistance	Ma, Mi, Mj,	Ma, Mi, Mj, Lt,	Ma, Mi, Mj, **Rs**,<br>Lt, TSWV,	Ma, Mi, Mj, **Rs**,<br>Lt, TSWV,

Abbreviation key:

Scientific Name	Common Name	Abbreviation
**Viral Pathogens**		
Tomato spotted wilt virus	Tomato spotted wilt	TSWV
**Bacterial Pathogens**		
Ralstonia solanacearum	Bacterial wilt	Rs
**Fungal Pathogens**		
Fusarium oxysporum f.sp. *lycopersici*	Fusarium wilt	Fol
Leveillula taurica	Powdery mildew	Lt
Verticillium albo-atrum	Verticillium wilt	Va
Verticillium dahliae	Verticillium wilt	Vd
**Nematode Pathogens**		
Meloidogyne arenaria	Root-knot	Ma
Meloidogyne incognita	Root-knot	Mi
Meloidogyne javanica	Root-knot	Mj

*The above information was obtained from the Stark Ayres online webpage: https://www.starkeayres.co.za/com_variety_docs/Tomatoes-Determinate-varieties-Crop-TableWebsite.pdf (accessed 27/05/2019).

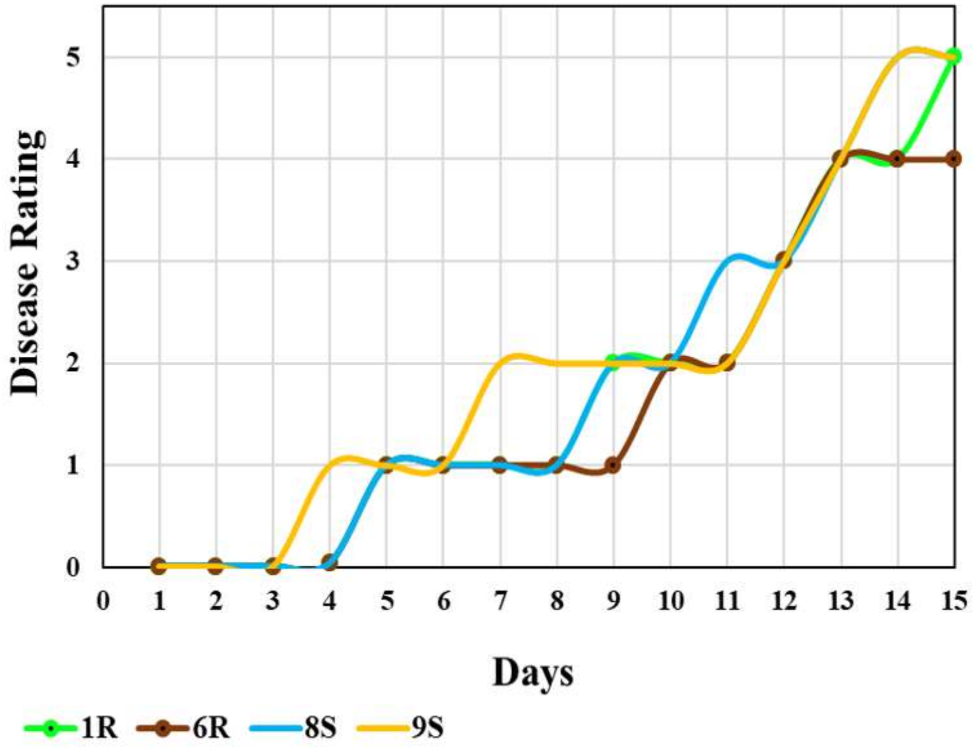

Figure S3. The disease severity index of the four tomato cultivars (1R, 6R, 8S, 9S) after infection with *Ralstonia solanacearum* and incubation over a time period of 15 days. A score of 0 indicated no leaves wilted and scores of 1–5 indicated 25%, 26–50%, 51–75%, 76–90% and 91–100% wilting respectively. The metabolic phenotypes were subsequently analysed and compared at 15 day.

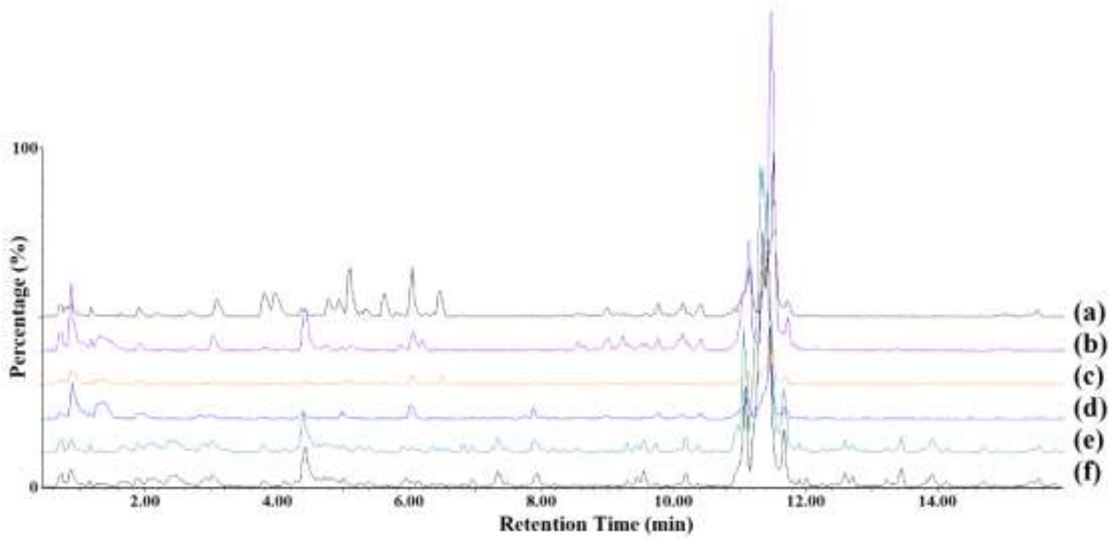

Figure S4. A representative UHPLC-MS base peak intensity (BPI) chromatogram (ESI(+) mode) overlay showing the metabolite profiles of tissues from the *Solanum lycopersicum* cultivar ‘1R’ before and after treatment with *R. solanacearum.* **(a)** The 1R treated leaf sample, **(b)** The 1R control leaf sample, **(c)** The treated 1R stem sample, **(d)** The 1R control stem sample, **(e)** The 1R treated root sample, **(f)** The 1R control root sample. The y-axis represents the relative abundance (%) of the metabolite fragments at their respective retention times (min). Throughout the article, the results obtained from ESI(–) mode were graphically presented in the figures due to many of the metabolites ionizing better in the negative mode.

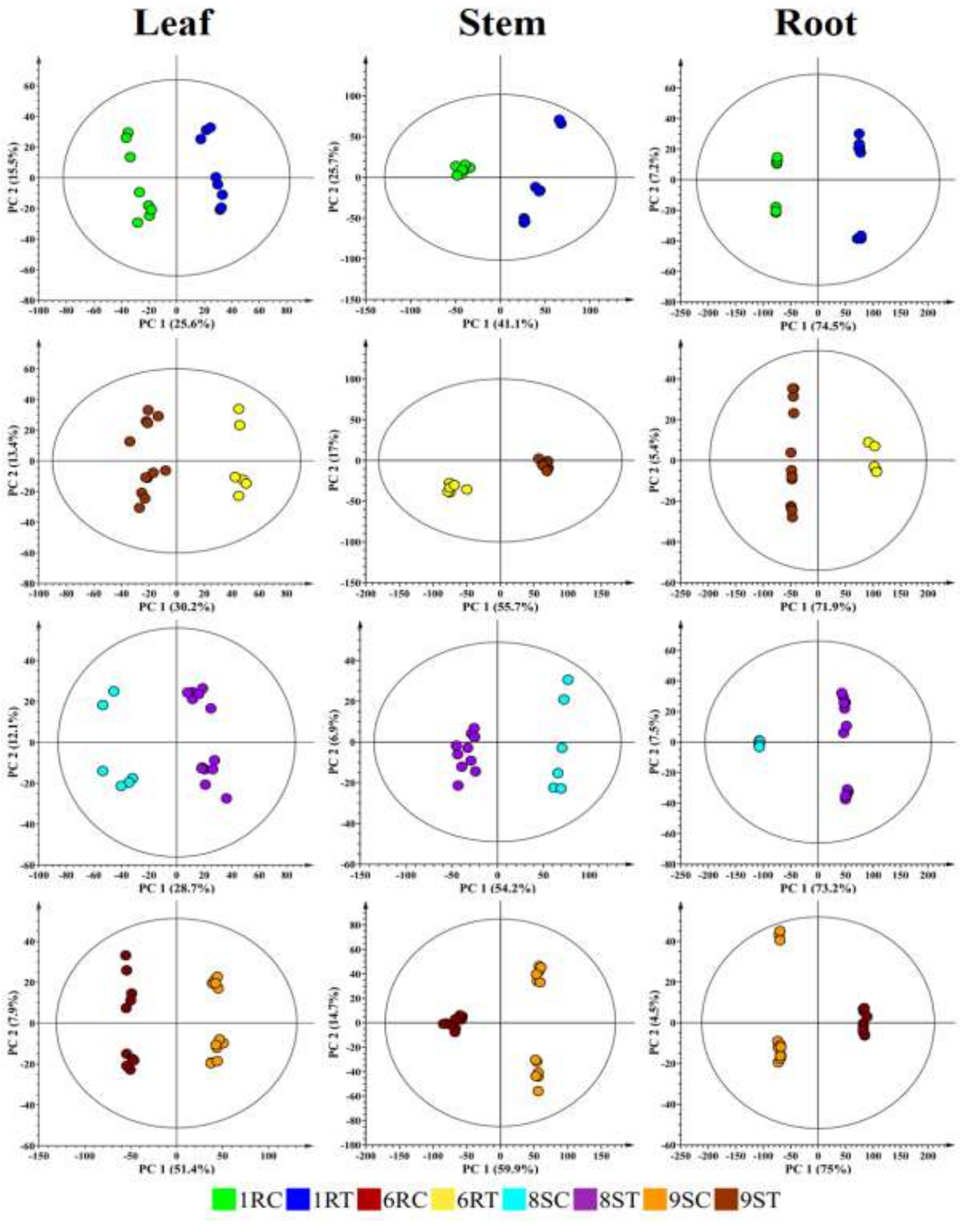

Figure S5. Computed PCA scores plots of the UHPLC-MS ESI(–) data of the extracts prepared from the leaf, stem and root tissues of four *Solanum lycopersicum* cvs. treated with *Ralstonia solanacearum*. The labels C and T refer to the control and treatment of the four cultivars (1R, 6R, 8S, and 9S). The ellipse on the score plots represents Hoteling's T2 with a 95% confidence interval.

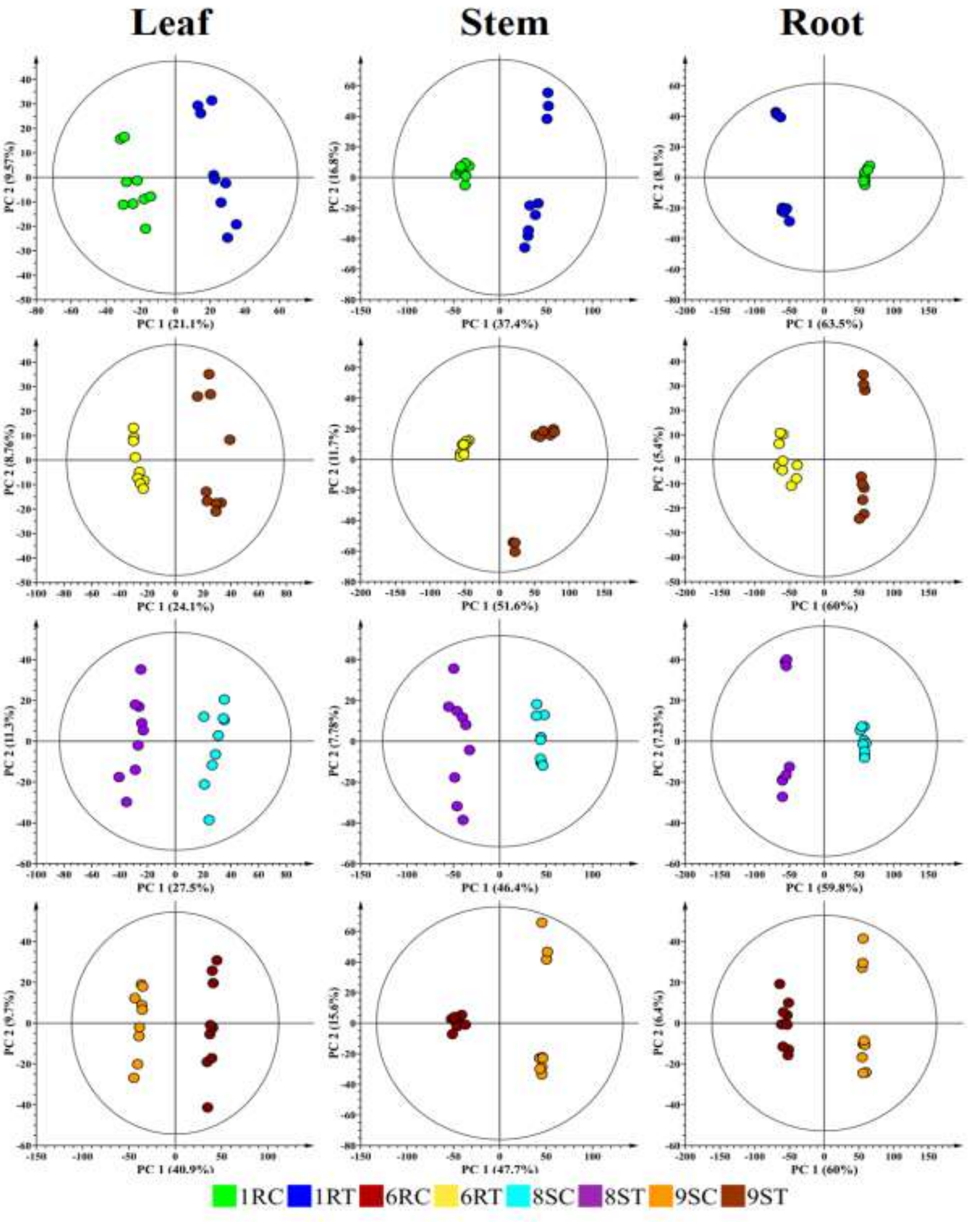

Figure S6. Computed PCA scores plots of the UHPLC-MS (ESI+) data of the extracts prepared from the leaf, stem and root tissues of four *Solanum lycopersicum* cvs. treated with *Ralstonia solanacearum*. The labels C and T refer to the control and treatment of the four cultivars (1R, 6R, 8S, and 9S). The ellipse on the score plots represents Hoteling's T2 with a 95% confidence interval.

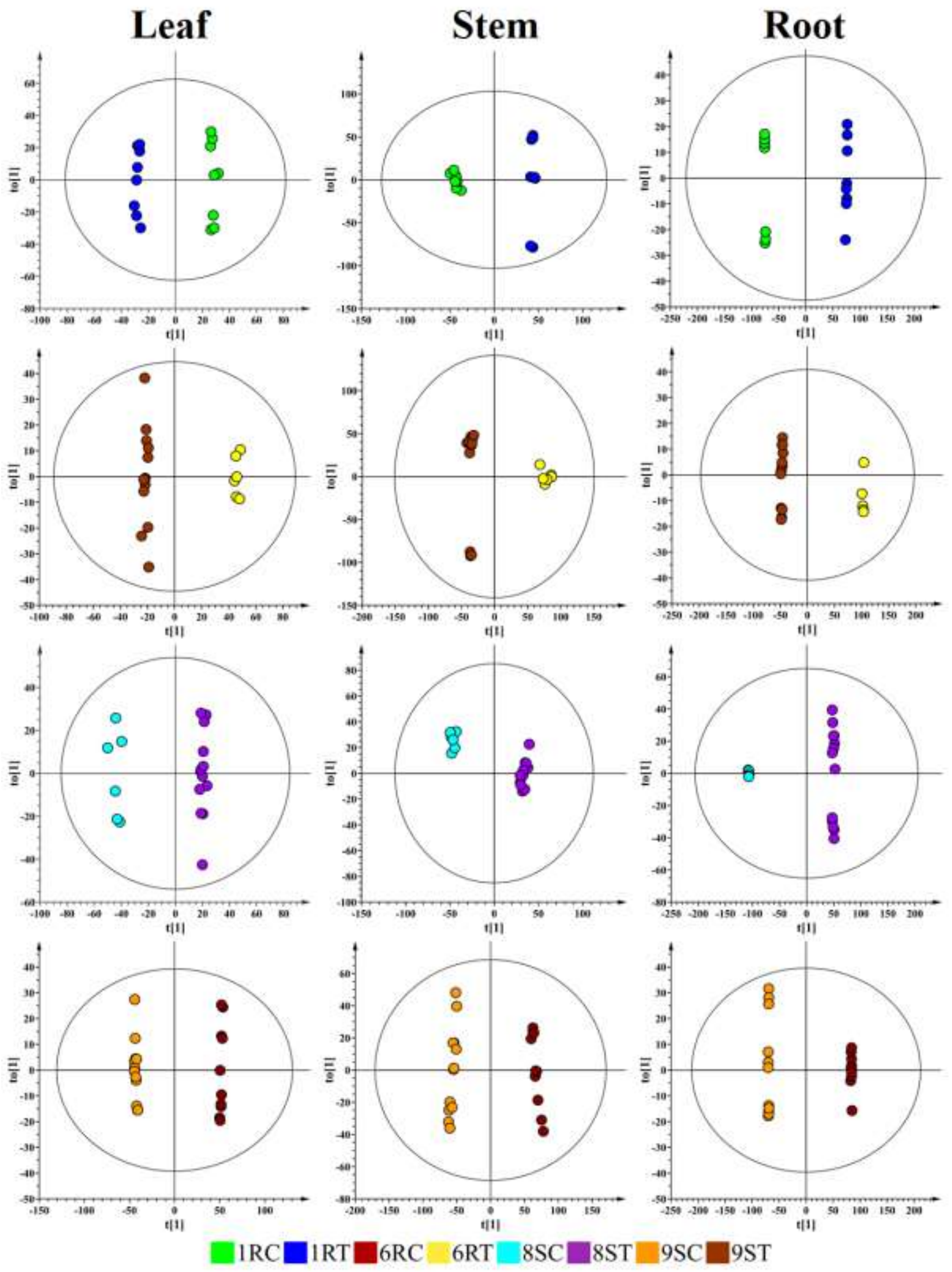

Figure S7. Computed OPLS-DA models for the ESI(–) data processing of leaf, stem and root tissue extracts of the four tomato cultivars (1R, 6R, 8S, and 9S). The labels C and T refer to the control and treatment of the four cultivars. The ellipse in each model represents Hoteling's T2 with a 95% confidence interval.

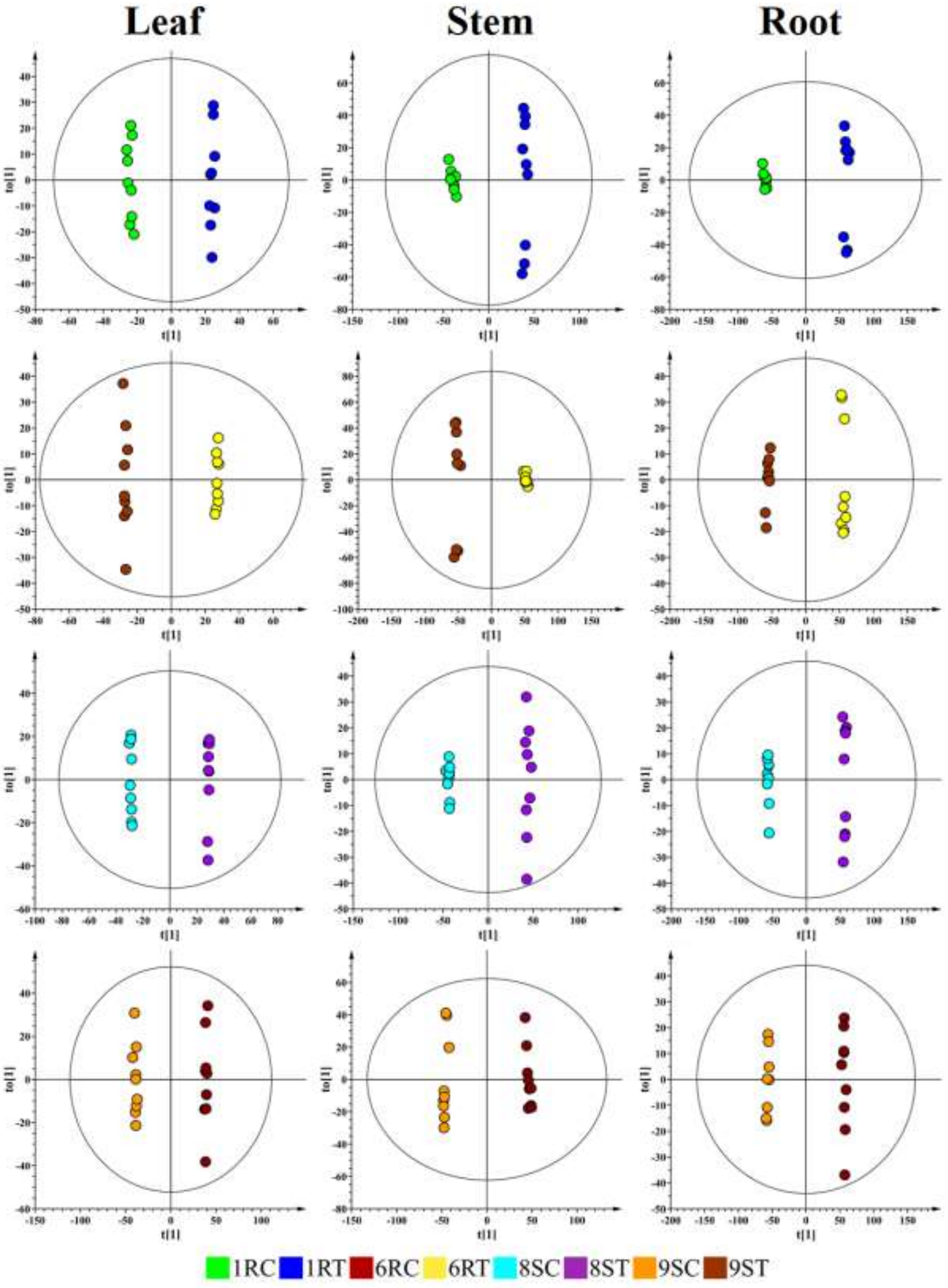

Figure S8. Computed OPLS-DA models for the ESI(+) data processing of leaf, stem and root tissue extracts of the four tomato cultivars (1R, 6R, 8S, and 9S). The labels C and T refer to the control and treatment of the four cultivars. The ellipse in each model represents Hoteling's T2 with a 95% confidence interval.

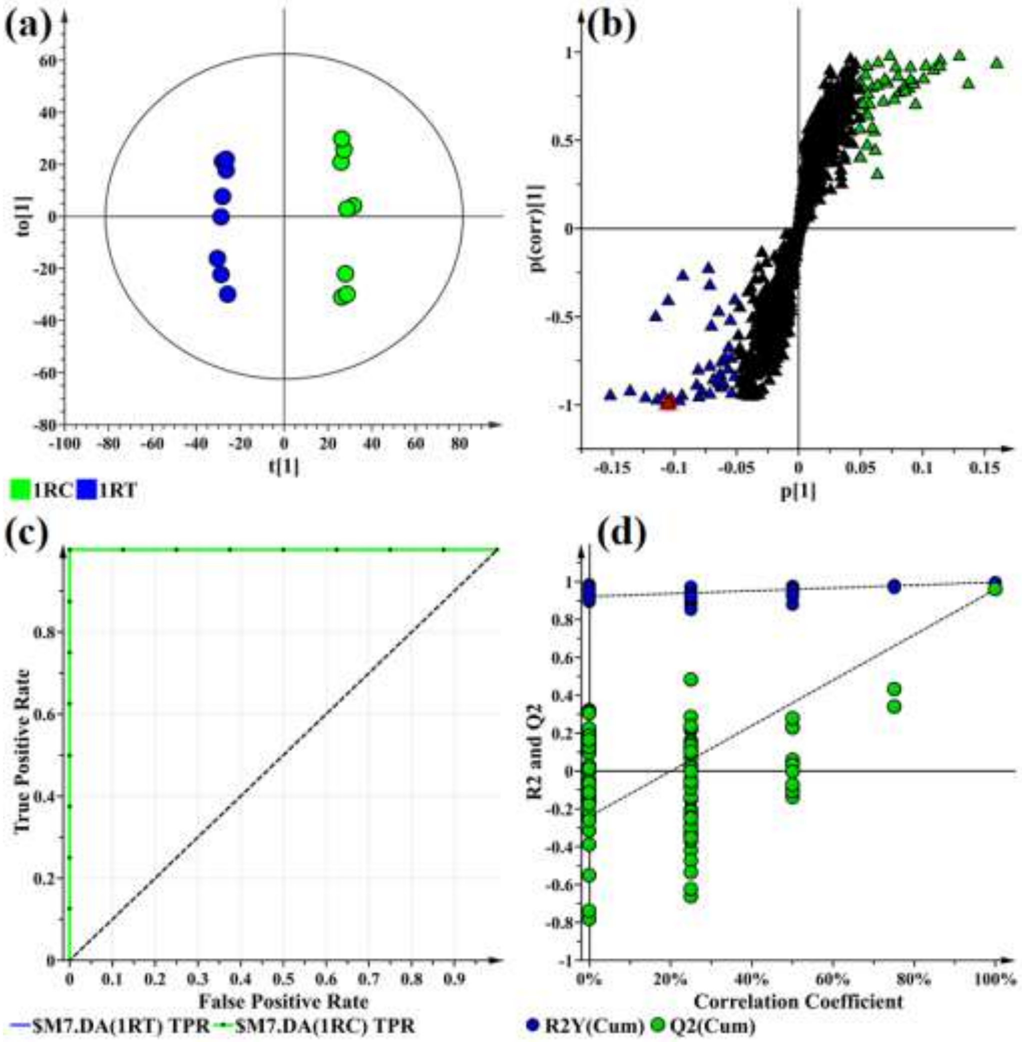

Figure S9. OPLS-DA model for the ESI(-) data processing of leaf tissue extracts of the tomato '1R' cv., control (1RC: Green) and treated (1RT: Blue). **(a)** An OPLS-DA plot showing the group separation, **(b)** The corresponding OPLS-DA loading S-plot. Relevant variables far out in the loadings S-plot (x,y > 0.05) were selected and represent possible discriminating variables. **(c)** A receiver operating characteristic (ROC) curve summarises the ability of a binary classifier (S-plot), with a classifier having a perfect discrimination producing a ROC curve that passes through the top left corner to indicate 100% sensitivity and specificity. **(d)** The response permutation test plot (n = 100) for the OPLS-DA model.

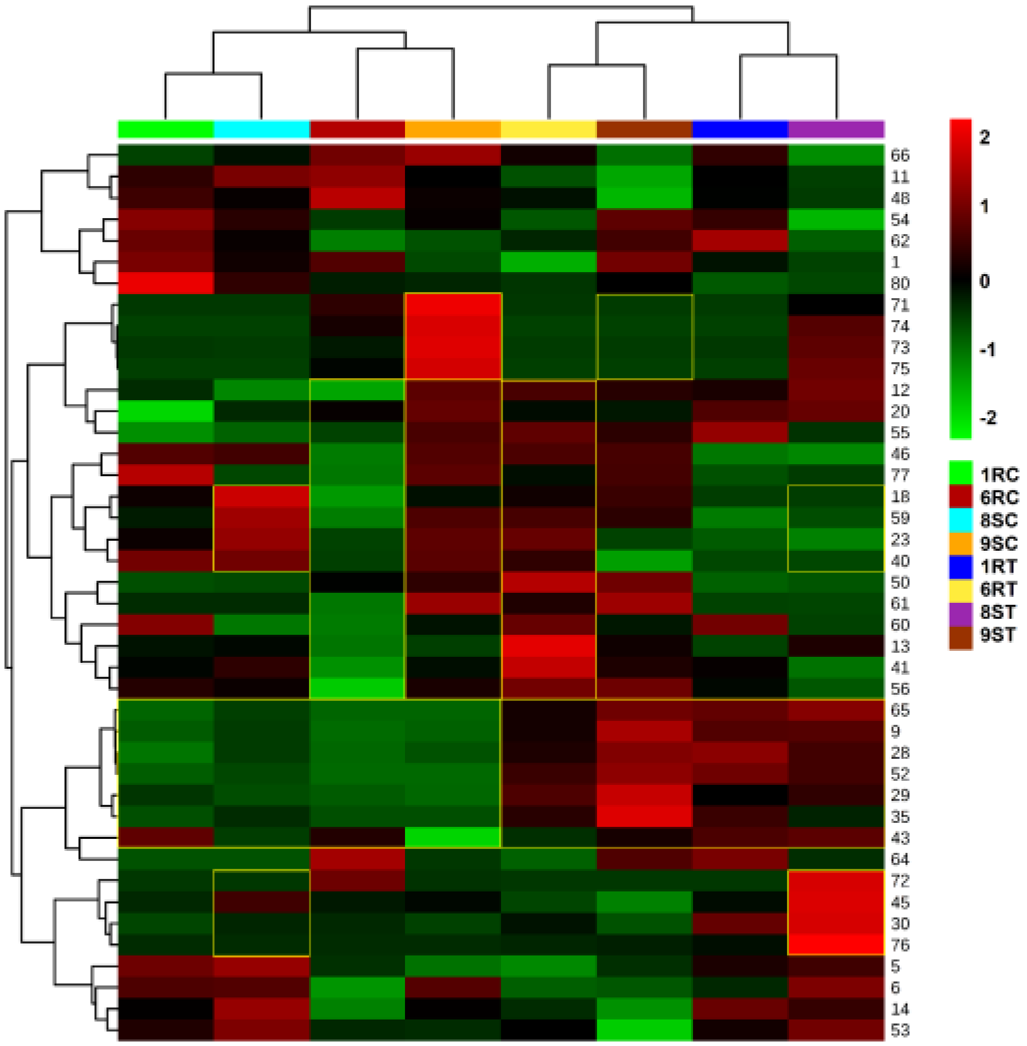

Figure S10. Heatmap analysis (Pearson distance and complete linkage rule applied) showing the individual fold changes of the 42 differential metabolite ions identified in the stem tissue between the four tomato (*Solanum lycopersicum*) cultivars upon treatment with *Ralstonia solanacearum*. Shown is a heatmap of the mean peak intensity of each annotated metabolite following normalization and Pareto scaling of the data. The colour scheme is noted in the legend above, indicating fold change increases (red), decreases (green) and significant changes between cv. conditions (yellow borders). Each row represents a discriminant metabolite feature provided in Table S2. The first four columns show the control tomato cultivars while the last four indicate the treated cultivars.

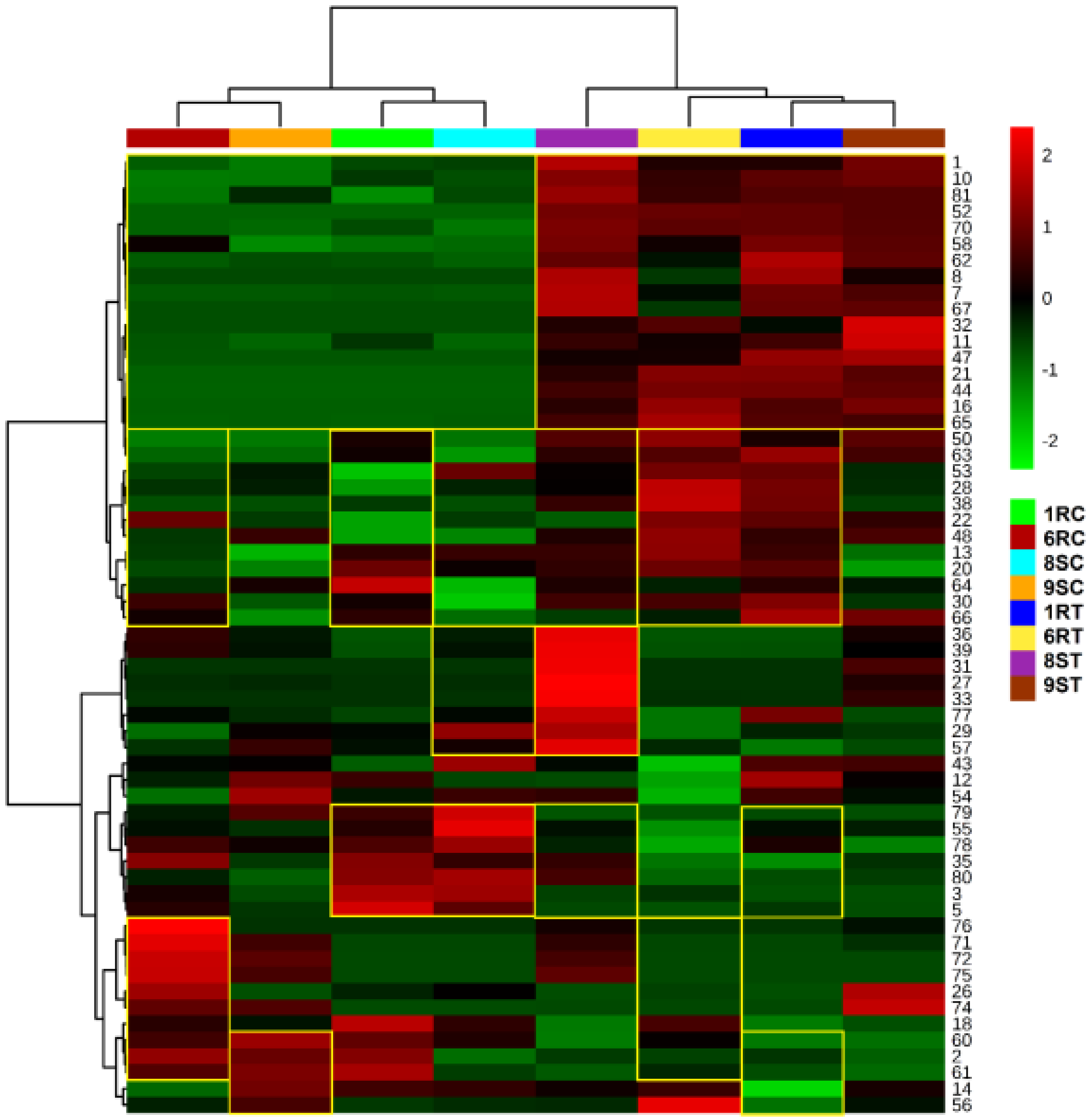

Figure S11. Heatmap analysis (Pearson distance and Ward's linkage rule applied) showing the individual fold changes of the 59 differential metabolite ions identified in the root tissue between the four tomato (*Solanum lycopersicum*) cultivars upon treatment with *Ralstonia solanacearum*. Shown is a heatmap of the mean peak intensity of each annotated metabolite following normalization and Pareto scaling of the data. The colour scheme is noted in the legend above, indicating fold change increases (red), decreases (green) and significant changes between cv. conditions (yellow borders). Each row represents a discriminant metabolite feature provided in Table S2. The first four columns show the control tomato cultivars while the last four indicate the treated cultivars.

Table S2. Annotated metabolites identified in tissues of *Ralstonia solanacearum*-infected tomato cultivars using UHLPC-QTOF-MS and chemometric analyses applied towards the identification of signatory/discriminatory biomarkers associated with the host response. The metabolite ions were annotated according to the Metabolomics Standards Initiative (MSI) level 2.

Peak[a]	Rt (min)	Observed mass (*m/z*)	Tentative compound identification	Chemical formula	Pubchem CID	Error (mDa)	Diagnostic *m/z* fragment ions[b]	Metabolite tissue presence	*p*-value (Corr)	Reference[c]
Hydroxycinnamic acid (HCA) derivatives										
**1**	0.87	341.104	Caffeoylglycoside (I)	$C_{15}H_{18}O_9$	5281761	8.2	179 $[M-H-hex]^-$<br>135 $[M-H-hex-COOH]^-$	L/S/R	9.409×10^{-05}	[1]
**2**	1.42	341.091	Caffeoylglycoside (II)	$C_{15}H_{18}O_{10}$	5281761	3.9	179 $[M-H-hex]^-$<br>135 $[M-H-hex-COOH]^-$	L/R	0.00146141	[1]
**3**	1.64	371.059	Caffeoylglucaric acid (I)	$C_{15}H_{16}O_{11}$	689043	-5.5	209 $[M-H-caff]^-$<br>191 $[quin-H]^-$<br>179 $[M-H-hex]^-$<br>147 $[M-H-hex-OH]^-$<br>135 $[caff-H-COOH]^-$	L/R	0.020125	[2,3]
**4**	2.07	371.054	Caffeoylglucaric acid (II)	$C_{15}H_{16}O_{11}$	689043	-6.8	210 $[M-H-caff]^-$<br>191 $[quin-H]^-$<br>179 $[M-H-hex]^-$<br>147 $[M-H-hex-OH]^-$<br>135 $[caf-H-COOH]^-$	L	0.0122359	[2,3]
**5**	2.83	353.082	*trans*-3-Caffeoylquinic acid	$C_{16}H_{18}O_9$	25210304	-3.7	191 $[quin-H]^-$<br>179 $[M-H-hex]^-$<br>135 $[caff-H-COOH]^-$	L/S/R	0.0327546	[4]
**6**	2.99	355.065	Feruloylglycoside (I)	$C_{16}H_{20}O_9$	13962928	-	341 $[M-H-CH_3]^-$<br>193 $[M-H-hex]^-$	L/S	4.25×10^{-05}	[5,6]
**7**	3.26	341.086	Caffeoylglycoside (III)	$C_{15}H_{18}O_9$	5281761	-2.4	179 $[M-H-hex]^-$<br>135 $[M-H-hex-COOH]^-$	L/R	0.756175	[1]

**8**	3.42	353.010	*cis*-3-Caffeoylquinic acid	$C_{16}H_{18}O_9$	1794427	-	707 [2M-H]$^-$<br>191 [quin -H]$^-$<br>179 [caff-H]$^-$<br>135 [caff-H-COOH]$^-$	L/R	0.00226525	[4]
**9**	3.98	385.072	Sinapoylglycoside (I)	$C_{17}H_{21}O_{10}$	5280406	-	223 [M-H-hex]$^-$<br>207 [M-H-hex-OH]$^-$<br>179 [M-H-2CH$_3$-hex-OH]$^-$	L/S	3.17 x 10^{-06}	[7]
**10**	4.08	431.150	Sinapoylglycoside (II)	$C_{17}H_{20}O_{11}$	5280406	-	385 [M-H-FA]$^-$<br>223 [M-H-hex]$^-$<br>207 [M-H-hex-OH]$^-$<br>179 [M-H-2CH$_3$-hex-OH]$^-$	L/R	0.000170975	[7]
**11**	4.10	367.102	*trans*-5-Feruloylquinic acid	$C_{17}H_{20}O_9$	101024370	-	191 [quin-H]$^-$<br>135 [M-H]$^-$	L/S/R	5.66 x 10^{-09}	[6]
**12**	4.41	353.083	*trans*-5-Caffeoylquinic acid	$C_{16}H_{18}O_9$	12310830	-4.5	191 [quin-H]$^-$<br>135 [caff-H-COOH]$^-$	L/S/R	0.0222839	[4]
**13**	5.07	355.100	Feruloylglycoside (II)	$C_{16}H_{20}O_9$	13962928	-4.2	341 [M-H-CH3]$^-$<br>193 [M-H-hex]$^-$	L/S/R	1.56 x 10^{-08}	[5,7]
**14**	5.21	385.109	Sinapoylglycoside (III)	$C_{17}H_{21}O_{10}$	5280406	-5	223 [M-H-hex]$^-$<br>207 [M-H-hex-OH]$^-$<br>179 [M-H-2CH$_3$-hex-OH]$^-$	L/S/R	4.22 x 10^{-05}	[7]
**15**	5.27	353.051	trans-4-Caffeoylquinic acid	$C_{16}H_{18}O_9$	9798666	-	191 [quin - H]$^-$<br>179 [caff-H]$^-$<br>173 [quin-H-H$_2$O]$^-$<br>135 [caff-H-COOH]$^-$	L	0.00105657	[4]
**16**	5.71	353.051	*cis*-5-Caffeoylquinic acid	$C_{16}H_{18}O_9$	1794425	-	191 [quin-H]$^-$<br>135 [M-H-COOH]$^-$	L/R	0.0420246	[4]
**17**	5.79	337.089	*trans*-5-Coumaroylquinic acid	$C_{16}H_{17}O_8$	90478782	-8	191 [quin-H]$^-$<br>163 [M-H-coumaric acid]-<br>119 [M-H]$^-$	L	3.84 x 10^{-06}	[5,8]

**18**	5.92	385.184	Feruloylglucaric acid (I)	$C_{16}H_{18}O_{11}$	445858		209 $[M-H-fer]^-$<br>193 $[fer-H]^-$<br>147 $[M-H-hex-OH]^-$	L/S/R	2.32 x 10^{-06}	[2]
**19**	6.15	343.100	Dihydrocaffeic acid hexoside	$C_{15}H_{20}O_9$	348154	-6.8	181 $[M-H]^-$<br>163 $[M-H]^-$<br>137 $[M-H]^-$<br>119 $[M-H]^-$	L	0.000535216	
**20**	6.36	367.102	*cis*-5-Feruloylquinic acid	$C_{17}H_{20}O_9$	101024370	-5.5	191 $[quin-H]^-$<br>135 $[M-H]^-$	L/S/R	0.018923	[6]
**21**	6.44	529.156	Feruloylquinic acid hexoside	$C_{23}H_{30}O_{14}$	-	1.3	367$[M-H-hex]^-$<br>191 $[quin-H]^-$	R	0.00115873	[9]
**22**	6.58	469.225	4-Hydroxycoumarin rhamnose hexoside (I)	$C_{21}H_{26}O_{12}$	54682930		504 $[M-H+Cl]^-$<br>439 $[M-H-CH_3-OH]^-$<br>307 $[M-H-hex]^-$<br>179 $[M-H-rham-hex+H_2O]^-$<br>161 $[hydroxycoumarin-H]^-$	L/R	0.000192581	
**23**	6.67	337.146	*cis*-5-Coumaroylquinic acid	$C_{16}H_{17}O_8$	6441280		191 $[quin-H]^-$<br>163 $[M-H-coum]^-$	L/S	3.84 x 10^{-06}	[5,8]
**24**	6.82	469.225	4-Hydroxycoumarin rhamnose hexoside (II)	$C_{21}H_{26}O_{12}$	54682930		504 $[M-H+Cl]^-$<br>439 $[M-H-CH_3-OH]^-$<br>307 $[M-H-hex]^-$<br>179 $[M-H-rham-hex+H_2O]^-$<br>161 $[hydroxycoumarin-H]^-$	L	1.27 x 10^{-08}	
**25**	10.41	591.171	Feruloyl sinapoyl glucaric acid	$C_{27}H_{28}O_{15}$	-	-2.9	397 $[M-H]^-$<br>385 $[M-H-223]^-$<br>223 $[sinapic acid-H]^-$<br>193 $[fer-H]^-$	L	5.04 x 10^{-06}	[2]
Hydroxycinnamic acid amides (HCA amides)/Phenylamides (PhA)										

**26**	2.32	251.1384	Caffeoyl putrescine	$C_{13}H_{19}N_2O_3$	5280559	-0.6	234 [M+H-OH]$^+$<br>145 [M+H-put-H_2O]$^+$<br>89 [M+H-caff]$^+$	L/R	3.10 x 10^{-06}	[1,6,7,10]
**27**	3.81	265.157	Feruloyl putrescine	$C_{14}H_{20}N_2O_3$	5281796	1.7	531 [2M+H]$^+$<br>177 [M+H-put]$^+$<br>145. 117 [M+H-$C_9H_9O_2$]$^+$<br>89 [M+H-fer]$^+$	L/R	0.00184852	[6,10]
**28**	4.91	351.127	*trans*-Feruloyl serotonin	$C_{20}H_{19}N_2O_4$	5969616	-2.5	337[M-H-CH_3]-<br>178 [M-H-CH3-$C_{10}H_{10}NO$]-<br>163 [M-H-fer]-	L/S/R	2.76 x 10^{-10}	[11]
**29**	5.08	411.184	Caffeoylputrescine glycoside	$C_{19}H_{27}N_2O_8$	129850233	2.8	321 [M-H]-<br>249 [M-H-hex]-<br>179 [caff-H]-<br>135 [caff-H-COOH]-	L/S/R	4.97 x 10^{-09}	[5]
**30**	5.82	351.126	*cis*-Feruloyl serotonin	$C_{20}H_{19}N_2O_4$	5969616	-7.7	337[M-H-CH_3]-<br>178 [M-H-CH3-$C_{10}H_{10}NO$]-<br>163 [M-H-fer]-	L/S/R	0.000253978	[11]
**31**	6.47	295.105	Sinapoyl putrescine	$C_{15}H_{22}NO_4$	-		279 [M+H-OH]$^+$<br>89 [M+H-sinapic acid]$^+$	L/R	0.00162172	[10]
**32**	7.79	444.165	Coumaroyltyramine glycoside	$C_{23}H_{26}NO_8$	5372945	6.7	444 [M-H]$^-$<br>282 [M-H-hex]$^-$	L/R	0.00792325	[5]
**33**	7.8	284.132	Coumaroyltyramine	$C_{17}H_{17}NO_3$	5372945	1.6	149 [M+H-C_8H_7O-OH]$^+$<br>136 [M+H-coum]$^+$	L/R	8.10 x 10^{-05}	[10,12]
**34**	7.96	300.127	Coumaroyl dopamine	$C_{17}H_{17}NO_4$	11630793		300 [M+H]$^+$	L	0.000425363	[13]

**35**	8.21	474.174	Feruloyltyramine glycoside	$C_{24}H_{28}NO_9$	-	-1.4	312 [M-H-hex]$^-$<br>178 [M-H]$^-$	L/S/R	1.18 x 10^{-12}	[5]
**36**	8.22	314.144	Feruloyl tyramine	$C_{18}H_{20}NO_4$	5280537	-0.6	177 [M+H-C_8H_8]$^+$<br>164 [M+H-$C_9H_9O_2$]$^+$<br>137 [M+H-fer]$^+$<br>121 [M+H-fer-OH]$^+$	L/R	7.77 x 10^{-15}	[7,10]
**37**	8.35	330.136	Feruloyl dopamine	$C_{18}H_{19}NO_5$	16119330		330 [M+H]+	L	1.19 x 10^{-08}	[13]
**38**	8.47	504.185	Feruloylmethoxytyramine glycoside	$C_{25}H_{30}NO_{10}$	5352115	4.2	342 [M-H]$^-$<br>327 [M-H]$^-$<br>273 [M-H]$^-$	L/R	6.07 x 10^{-11}	[5]
**39**	8.56	498.1667	Diferuloyl spermidine	$C_{27}H_{37}N_3O_6$	-		498 [M+H]$^+$	L/R	2.47 x 10^{-09}	[14]
Flavonoids										
**40**	4.96	771.202	Quercitin dihexose deoxyhexoside	$C_{33}H_{40}O_{21}$	44259182	-0.6	609 [M-H]$^-$<br>463 [M-H]$^-$<br>301 [quercetin-H]$^-$<br>[M-hex]$^-$	L/S	0.00386845	[9] X
**41**	7.16	741.188	Quercitin hexose deoxyhexose pentose	$C_{32}H_{38}O_{20}$	44259292	1.6	609 [M-H-pent]$^-$<br>463 [M-H-deoxyhex-pent]$^-$<br>301 [quercetin-H]$^-$<br>[M-hex]$^-$	L/S	0.0139979	[3]
**42**	7.87	725.197	Quercitin rutinoside pentoside	$C_{32}H_{38}O_{19}$	-	-3.4	609 [M-H]-<br>301 [quercetin-H]$^-$<br>[M-hex]$^-$	L	0.00568073	X

**43**	7.90	609.146	Rutin	$C_{27}H_{30}O_{16}$	5280805	6.4	301 [quercetin-H]$^-$	L/S/R	0.00260765	[1,3]
**44**	8.02	449.188	Eriodictyol glycoside	$C_{21}H_{22}O_{11}$	13254473	-	287 [eriodictyl - H]$^-$<br>[M-hex]$^-$	L/R	0.00497438	[9,15]
**45**	8.17	463.089	Quercetin glycoside	$C_{21}H_{20}O_{12}$	44259136	-2.9	301 [quercetin-H]$^-$<br>[M-hex]$^-$<br>271 [M-H]$^-$	L/S	5.29 x 10^{-09}	[3]
**46**	8.83	593.149	Kaempferol 3-rutinoside	$C_{27}H_{30}O_{15}$	71600048	-1.7	285 [M-H-2hex]$^-$	L/S	7.67 x 10^{-08}	[3,9]
**47**	9.12	447.218	Kaempferol-glycoside	$C_{21}H_{20}O_{11}$	5282102	4	285 [kaempferol-H]$^-$<br>[M-hex]$^-$	L/R	0.0456161	[3,9]
Organic acids										
**48**	0.97	133.011	Malic acid	$C_4H_5O_5$	525	-7	115 [M-H-H_2O]$^-$	L/S/R	5.30 x 10^{-07}	[15]
**49**	0.98	115.000	Fumaric acid	$C_4H_4O_4$	444972	-1	71 [M-H-COOH]$^-$<br>58 [M-H-$C_2H_2O_2$]-	L	4.62 x 10^{-11}	[15]
**50**	2.06	191.026	Citric acid	$C_6H_8O_7$	311	-7.2	173 [M-H-H_2O]$^-$<br>111 [M-H-CO_2-2H_2O]$^-$	L/S/R	0.00193365	[3,15,16]
**51**	3.99	191.016	Isocitric acid	$C_6H_8O_7$	1198	-3.1	173 [M-H-H_2O]-<br>111 [M-H-CO_2-2H_2O]-	L	0.000923945	[15,16]
**52**	4.77	323.131	Citrate pentoside	$C_{11}H_{16}O_{11}$	-		191 [M-H-pen]$^-$<br>175 [M-H-pent-OH]$^-$	L/S/R	1.24 x 10^{-05}	
**53**	5.07	175.036	Ascorbic Acid	$C_6H_8O_6$	54670067	9.4	115 [M-H-$C_2H_5O_2$]$^-$	L/S/R	0.00139602	[15]
Amino acids & derivatives										
**54**	0.85	146.043	Glutamic acid	$C_5H_8NO_4$	33032	-6.7	131 [M-H-NH_2]$^-$<br>128 M-H-OH]$^-$<br>102 [M-H-COOH]$^-$	L/S/R	0.000502658	[15]
**55**	1.17	128.032	Pyroglutamic acid	$C_5H_6NO_3$	7405	-2.7	84 [M-H-COOH]$^-$	L/S/R	1.08 x 10^{-06}	[15]
**56**	1.90	166.000	Phenylalanine	$C_9H_{12}NO_2$	6140	-9.4	149 [M+H-NH_3]$^-$<br>121 [M+H-COOH]$^+$	L/S/R	0.000414821	[1,3]
**57**	2.3	218.1	Panthothenic acid	$C_9H_{17}NO_5$	6613	-5.8	146 [M-H-$C_3H_5O_2$]$^-$	L/R	0.00186588	[15]
**58**	5.92	172.095	Acetyl leucine/isoleucine	$C_8H_{14}NO_3$	70912	-6.7	-	L/R	4.64 x 10^{-07}	

**59**	7.51	245.088	Acetyl Tryptophan	$C_{13}H_{14}N_2O_3$	2002	-7.4	203 $[M-H-C_2H_2O]^-$<br>142 $[M-H-NH_3-COOH-C_2H_2O]^-$<br>116 $[M-H-C_5H_8NO_3]^-$	L/S	1.74 x 10^{-08}	[1,3,15]
**60**	7.71	219.078	Hydroxytryptophan	$C_{13}H_{11}N_2O_4$	144		203 $[M-H-OH]^-$<br>146 $[M-H-C_2H_4NO_2]^-$<br>116 $[M-H-C_3H_6NO_2]^-$	L/S/R	0.00386036	
Hydroxybenzoic acid (HBA) derivatives										
**61**	3.00	153.018	Dihydroxybenzoic acid	$C_7H_40_4$	9338	-6.4	137 [M-H-OH]-<br>109 [M-COOH]-	L/S/R	0.0531	[1,17] X
**62**	3.31	285.055	Dihydroxybenzoic acid pentose	$C_{12}H_{13}O_8$	-	-4.2	153 $[M-H-pent]^-$<br>137 $[M-H-OH]^-$<br>109 $[M-COOH]^-$	L/S/R	9.13 x 10^{-10}	[1]
**63**	3.75	137.021	Salicylic acid	$C_7H_6O_3$	338	-9.8	109 $[M-H-OH]^-$<br>93 $[M-H-COOH]^-$	L/R	0.0005	X
**64**	4.99	401.142	Benzyl alcohol hexose-pentose	$C_{18}H_{25}O_{10}$	244	-2.4	269 $[M-H-pent]^-$<br>107 $[M-H-hex-pent]^-$	L/S/R	0.002	[1,3]
**65**	5.59	445.138	Methylsalicylate hexose pentose	$C_{19}H_{26}O_{12}$	11279		283 $[M-H-hex]^-$<br>269 $[M-H-hex-CH_3]^-$<br>299 $[M-H-pent-CH_3]^-$	L/S/R	6.32 x 10^{-06}	[3]
**66**	6.02	381.173	3-Methylbutyl 6-O-D-apio-b- Dfuranosyl-b- D-glucopyranoside	$C_{16}H_{30}O_{10}$	-	-2.5	423 [M-H-C2H3O]$^-$<br>249 [M-H-132 Da]$^-$<br>179 [M-H-202 Da]$^-$	L/S/R	3.01 x 10^{-09}	[15]
**67**	6.16	299.076	Salicylic acid glycoside	$C_{13}H_{16}O_8$	49859589	-10	137 $[M-H-hex]^-$	L/R	0.0033	[15]
**68**	6.34	435.089	Hydroxybenzoyl dihydroxybenzoic acid hexoside	$C_{20}H_{19}O_{11}$	-	2.5	315 [M-H-120 Da]$^-$<br>297 [M-H-138 Da]$^-$<br>153 $[M-H-hex]^-$<br>137 [M-H-hex-C6H5COOH]$^-$	L	0.0454	
**69**	7.36	315.063	Dihydroxybenzoic acid hexoside	$C_{13}H_{15}O_9$	78522		153 $[M-H-hex]^-$<br>109 $[M-H-hex-COOH]^-$	L	4.48 x 10^{-10}	[2]

**70**	7.68	425.2	Absisic acid hexose ester	$C_{21}H_{29}O_9$	46173811	4.2	409 $[M-H-OH]^-$<br>263 $[M-H-hex]^-$<br>153 $[M-H-C_6H_7O-hex]^-$	L/R	0.00308631	[15]
Steroidal glycoalkaloids (SGAs)										
**71**	10.18	1050.548	Hydroxytomatine	$C_{50}H_{83}NO_{22}$	-		1164 [M+H+FA+3Na]+<br>1096 $[M+H+FA]^+$<br>578 $[M+H-2hex-pent]^+$<br>416 $[tomatidene +H]^+$	L/S/R	0.003905	[3,18]
**72**	11.07	1032.540	Dehydrotomatine	$C_{50}H_{81}NO_{21}$	101920881	-7.9	1076 $[M+H+FA]^+$<br>576 $[M+H-2hex-pent]^+$<br>527 $[M+H+Na]^+$<br>414 $[dehydrotomatidene+H]^+$	L/S/R	9.59×10^{-05}	[18,19]
**73**	11.29	1092.560	Lycoperoside A/B/C	$C_{52}H_{85}NO_{23}$	131751568	-2.3	960 $[M+H-pent]^+$	L/S	7.09×10^{-05}	X
**74**	11.37	1034.550	α-Tomatine (I)	$C_{50}H_{83}NO_{21}$	28523	-5.1	740 $[M+H-hex-pent]^+$<br>578 $[M+H-2hex-pent]^+$<br>528 $[M+H+Na]^{2+}$<br>416 $[tomatidene+H]^+$	L/S/R	0.00346284	[3,19]
**75**	11.66	1034.543	α-Tomatine (II)	$C_{50}H_{83}NO_{21}$	28523	-6.4	740 $[M+H-hex-pent]^+$<br>578 $[M+H-2hex-pent]^+$<br>528 $[M+H+Na]^{2+}$<br>416 $[tomatidene+H]^+$	L/S/R	0.000744116	[3]
**76**	11.80	1004.540	Tomatidine dihexoside dipentoside	$C_{49}H_{81}NO_{20}$	65576	-3.4	870 [M-H]-<br>740 [M-H-hex-pent]-<br>578 [M-H-2pent-hex]-<br>416 [tomatidene-H]-	L/S/R	0.00085934	[3,18]
Fatty acids										
**77**	13.93	327.213	Trihydroxy-octadecadienoic acid (I)	$C_{18}H_{31}O_5$	129669152	-0.5	309 $[M-H-H_2O]^-$	L/S/R	7.10×10^{-05}	[3]
**78**	14.36	242.175	3-Amino-13-oxo-tridecanoic acid	$C_{13}H_{24}NO_3$	5182019	1.2	225 $[M-H-NH_3]^-$	L/R	3.30×10^{-05}	[15]

**79**	14.91	327.217	Trihydroxy-octadecadienoic acid (II)	$C_{18}H_{31}O_5$	129669152	0.6	-	L//R	9.30 x 10^{-05}	[3]
**80**	15.01	329.227	Hydroxyoctadecanedioic acid (I)	$C_{18}H_{33}O_5$	23052243	-8.7	271 $[M-H-58]^-$<br>171 $[M-H-158]^-$	S/R	0.00237209	[1]
**81**	16.21	329.232	Hydroxyoctadecanedioic acid (II)	$C_{18}H_{33}O_5$	23052243	-3	271 $[M-H-58]^-$<br>171 $[M-H-158]^-$	L/R	8.77 x 10^{-09}	[1]

[a]Peak numbers assigned based on each compound class elution order.

[b]hex, loss of hexose moiety (-162u); pent, loss of pentose moiety (-132u); rham, loss of rhamnose moiety (-147u); caff, caffeic acid moiety; fer, ferulic acid moiety; coum, coumaric acid moiety; put, putrescine moiety; quin, quinic acid moiety; FA, formic acid adduct (- 46u).

[c] References indicated with an “X” can be found on the tomato metabolome database (MoTo) http://www.ab.wur.nl/moto/

References

1. Cichon, M.J.; Riedl, K.M.; Schwartz, S.J. A metabolomic evaluation of the phytochemical composition of tomato juices being used in human clinical trials. *Food Chem.* **2017**, *228*, 270–278.
2. Nguyen, T.-K.-O.; Jamali, A.; Grand, E.; Morreel, K.; Marcelo, P.; Gontier, E.; Dauwe, R. Phenylpropanoid profiling reveals a class of hydroxycinnamoyl glucaric acid conjugates in *Isatis tinctoria* leaves. *Phytochemistry* **2017**, *144*, 127–140.
3. Roldan, M.V.G.; Engel, B.; De Vos, R.C.H.; Vereijken, P.; Astola, L.; Groenenboom, M.; Van De Geest, H.; Bovy, A.; Molenaar, J.; Van Eeuwijk, F.; et al. Metabolomics reveals organ-specific metabolic rearrangements during early tomato seedling development. *Metabolomics* **2014**, *10*, 958–974.
4. Ncube, E.N.; Mhlongo, M.I.; Piater, L.A.; Steenkamp, P.A.; Dubery, I.A.; Madala, N.E. Analyses of chlorogenic acids and related cinnamic acid derivatives from *Nicotiana tabacum* tissues with the aid of UPLC-QTOF-MS/MS based on the in-source collision-induced dissociation method. *Chem. Cent. J.* **2014**, *8*, 66.
5. Narváez-Cuenca, C.-E.; Vincken, J.-P.; Zheng, C.; Gruppen, H. Diversity of (dihydro) hydroxycinnamic acid conjugates in Colombian potato tubers. *Food Chem.* **2013**, *139*, 1087–1097.
6. Jaiswal, R.; Müller, H.; Müller, A.; Karar, M.G.E.; Kuhnert, N. Identification and characterization of chlorogenic acids, chlorogenic acid glycosides and flavonoids from *Lonicera henryi* L. (Caprifoliaceae) leaves by LC-MSn. *Phytochemistry* **2014**, *108*, 252–263, doi:10.1016/j.phytochem.2014.08.023.
7. Jáuregui, O.; Medina-Remón, A.; Andrés-Lacueva, C.; Vallverdú-Queralt, A.; Medina-Remón, A.; Andres-Lacueva, C.; Lamuela-Raventós, R.M.; Vallverdú-Queralt, A.; Medina-Remón, A.; Andrés-Lacueva, C.; et al. Improved characterization of tomato polyphenols using liquid chromatography/electrospray ionization linear ion trap quadrupole Orbitrap mass spectrometry and liquid chromatography/electrospray ionization tandem mass spectrometry. *Rapid Commun. Mass Spectrom.* **2010**, *24*, 2986–2992.
8. Voynikov, Y.; Zheleva-Dimitrova, D.; Gevrenova, R.; Lozanov, V.; Zaharieva, M.M.; Tsvetkova, I.; Najdenski, H.; Yagi, S.; Almoulah, N.F.; Momekov, G. Hydroxycinnamic acid amide profile of *Solanum schimperianum* Hochst by UPLC-HRMS. *Int. J. Mass Spectrom.* **2016**, *408*, 42–50.
9. Ncube, E.N.; Steenkamp, P.A.; Madala, N.E.; Dubery, I.A. Stimulatory effects of acibenzolar-s-methyl on chlorogenic acids biosynthesis in *Centella asiatica* cells. *Front. Plant Sci.* **2016**, *7*, 1469.
10. Dastmalchi, K.; Cai, Q.; Zhou, K.; Huang, W.; Serra, O.; Stark, R.E. Solving the jigsaw puzzle of wound-healing potato cultivars: metabolite profiling and antioxidant activity of polar extracts. *J. Agric. Food Chem.* **2014**, *62*, 7963–7975.
11. Masike, K.; Mhlongo, M.I.; Mudau, S.P.; Nobela, O.; Ncube, E.N.; Tugizimana, F.; George, M.J.; Madala, N.E. Highlighting mass spectrometric fragmentation differences and similarities between hydroxycinnamoyl-quinic acids and hydroxycinnamoyl-isocitric acids. *Chem. Cent. J.* **2017**, *11*, 29.
12. Kang, J.; Price, W.E.; Ashton, J.; Tapsell, L.C.; Johnson, S. Identification and characterization of phenolic compounds in hydromethanolic extracts of sorghum wholegrains by LC-ESI-MSn. *Food Chem.* **2016**, *211*, 215–226.
13. Itkin, M.; Rogachev, I.; Alkan, N.; Rosenberg, T.; Malitsky, S.; Masini, L.; Meir, S.; Iijima, Y.; Aoki, K.; De Vos, R.; et al. GLYCOALKALOID METABOLISM1 is required for steroidal alkaloid glycosylation and prevention of phytotoxicity in tomato. *Plant Cell* **2011**, *23*, 4507–4525.
14. Cataldi, T.R.I.; Lelario, F.; Bufo, S.A. Analysis of tomato glycoalkaloids by liquid chromatography coupled with electrospray ionization tandem mass spectrometry. *Rapid Commun. Mass Spectrom.* **2005**, *19*, 3103–3110.
